# Supplementary material for: Chromoanagenesis Landscape in 10,000 TCGA Patients
Source: Cancers (Basel). 2021 Aug 20;13(16):4197. doi: 10.3390/cancers13164197 (PMC8392194; doi:10.3390/cancers13164197)
Supplement: Supplementary file 1 [file cancers-13-04197-s001.zip › Supplemental figures.pdf]

Supplementary Figures

Chromoanagenesis Landscape in 10,000 TCGA Patients

Roni Rasnic [1,*] and Michal Linial [2]

[1] The Rachel and Selim Benin School of Computer Science and Engineering, The Hebrew University of Jerusalem, Jerusalem 9190401, Israel
[2] Department of Biological Chemistry, Institute of Life Sciences, The Hebrew University of Jerusalem, Jerusalem 9190401, Israel; michall@mail.huji.ac.il
* Correspondence: roni.rasnic@mail.huji.ac.il

Figure S1. CNV frequency in the PCAWG-TCGA cohort

(a) Box plot of number of deleted genes for each chromoanagenesis subtype. **(b)** Box plot of number of amplified genes for each chromoanagenesis subtype.

a

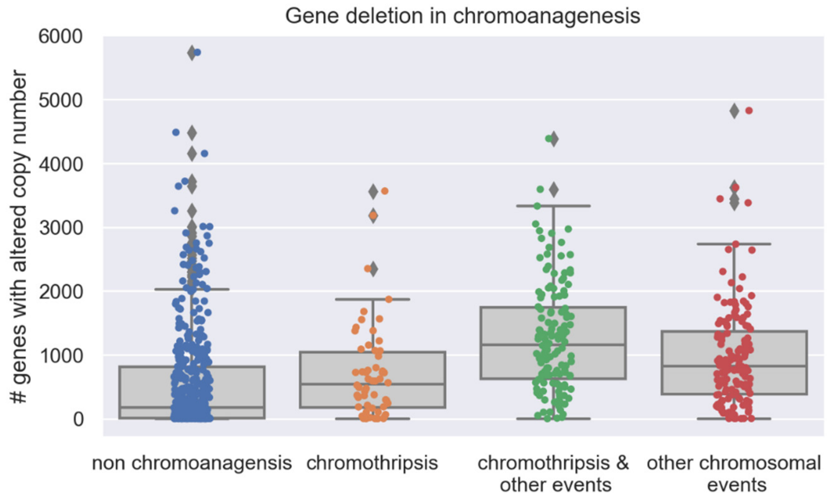

b

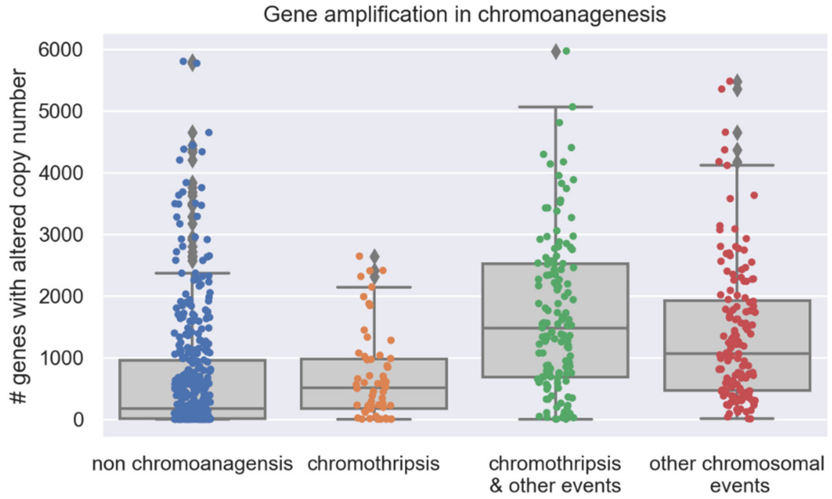

Figure S2. BLCA Manhattan plots

Genic Manhattan plot over Fisher's exact test p-values between BLCA chromoanagenesis samples and non-chromoanagenesis samples. **(a)** Manhattan CNA (deletion or amplification) plot. **(b)** Manhattan Deletion plot. **(c)** Manhattan Amplification plot.

a

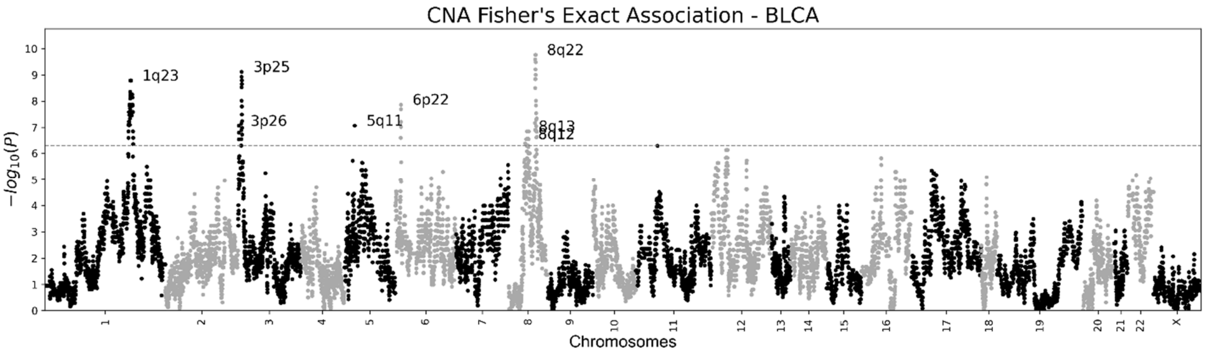

b

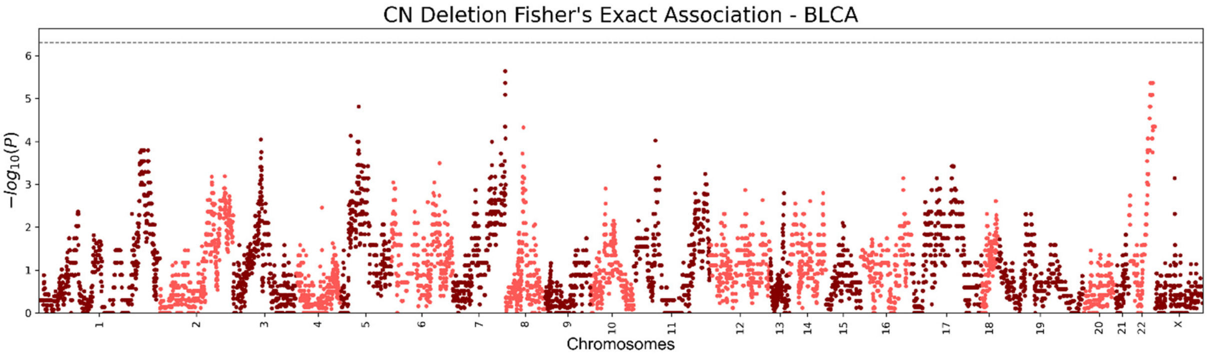

C

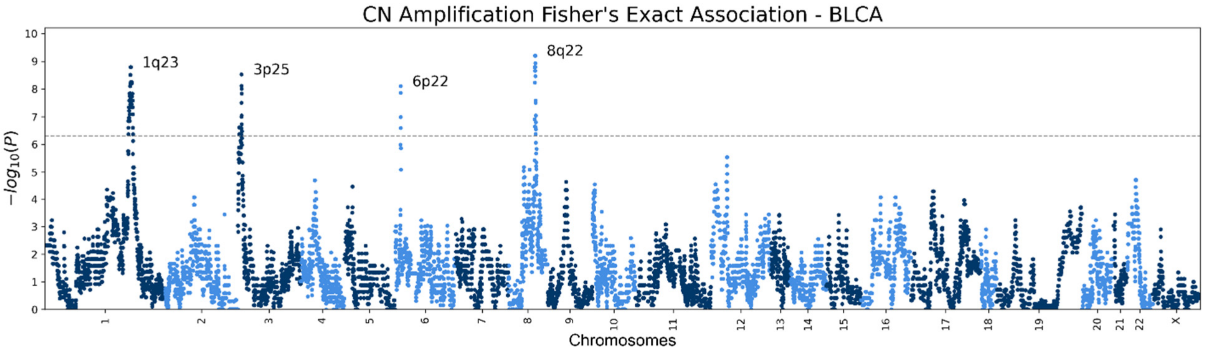

Figure S3. BRCA Manhattan plots

Genic Manhattan plot over Fisher's exact test p-values between BRCA chromoanagenesis samples and non-chromoanagenesis samples. **(a)** Manhattan CNA (deletion or amplification) plot. **(b)** Manhattan Deletion plot. **(c)** Manhattan Amplification plot.

a

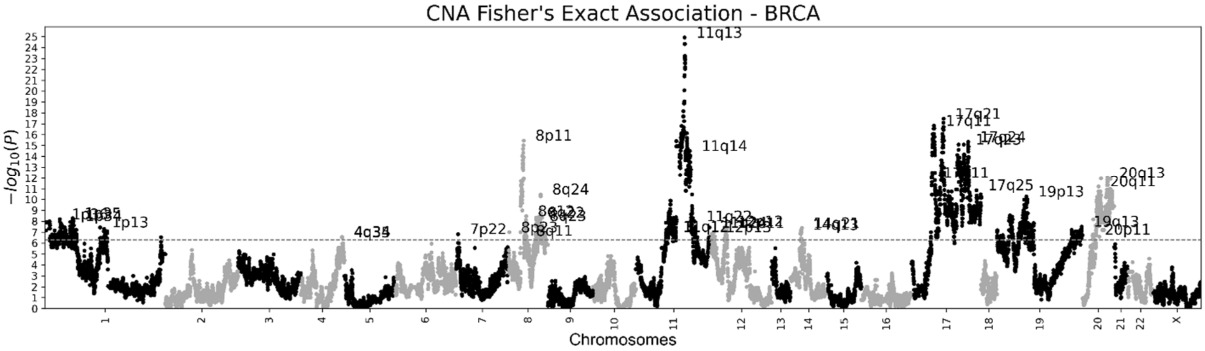

b

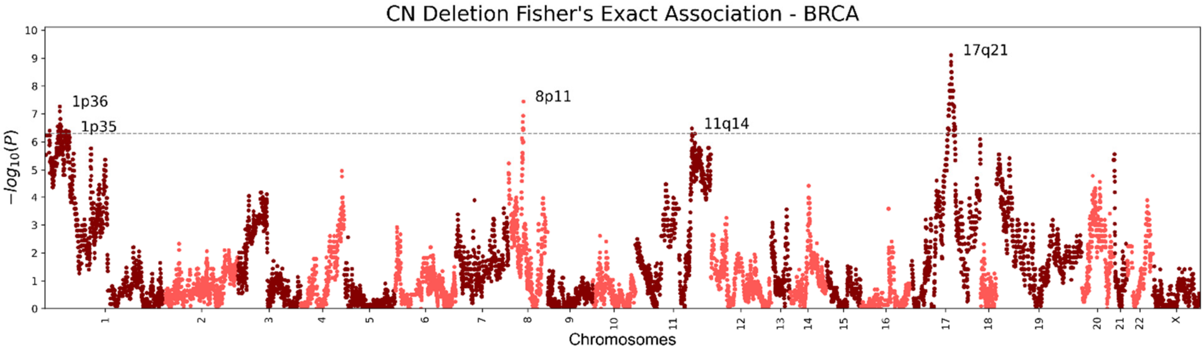

c

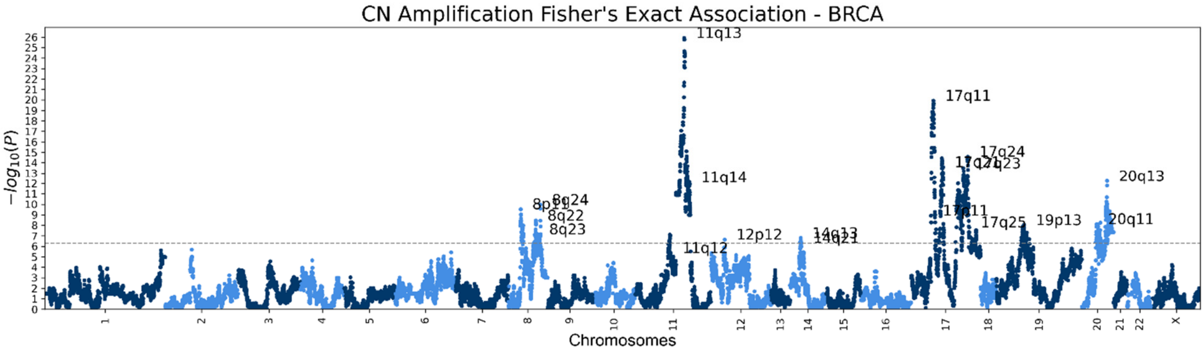

Figure S4. CESC Manhattan plots

Genic Manhattan plot over Fisher’s exact test p-values between CESC chromoanagenesis samples and non-chromoanagenesis samples. **(a)** Manhattan CNA (deletion or amplification) plot. **(b)** Manhattan Deletion plot. **(c)** Manhattan Amplification plot.

a

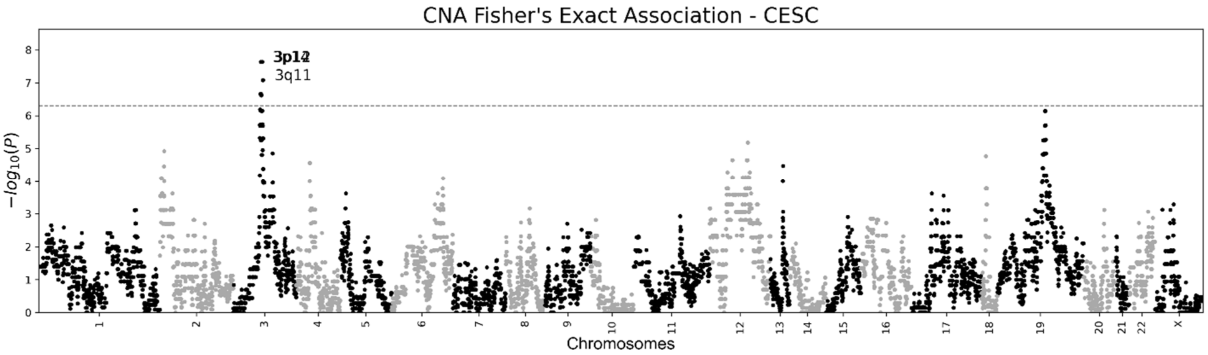

b

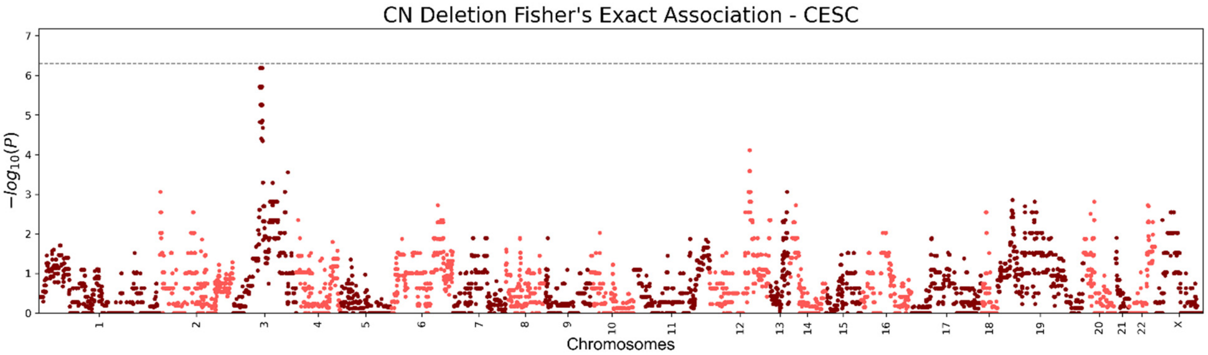

c

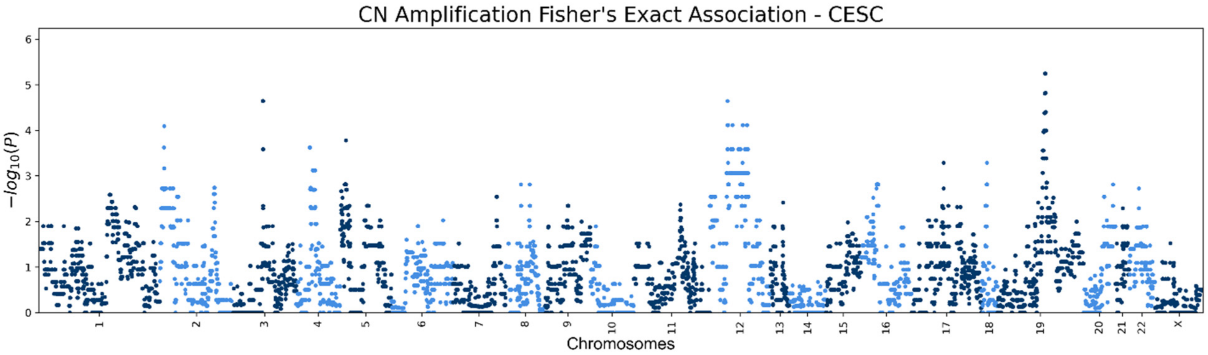

Figure S5. COAD Manhattan plots

Genic Manhattan plot over Fisher's exact test p-values between COAD chromoanagenesis samples and non-chromoanagenesis samples. **(a)** Manhattan CNA (deletion or amplification) plot. **(b)** Manhattan Deletion plot. **(c)** Manhattan Amplification plot.

a

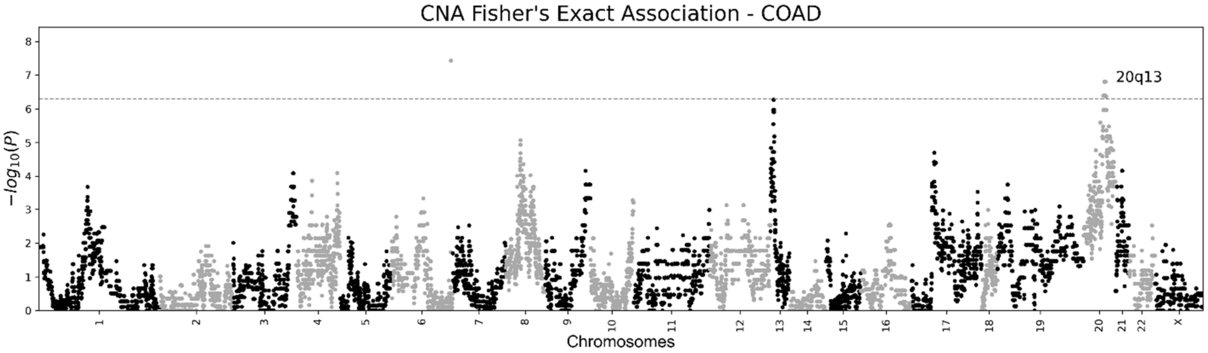

b

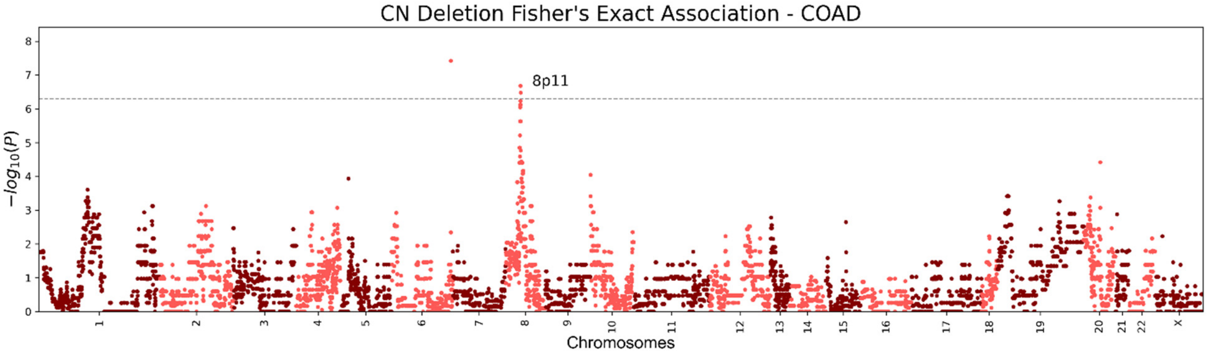

c

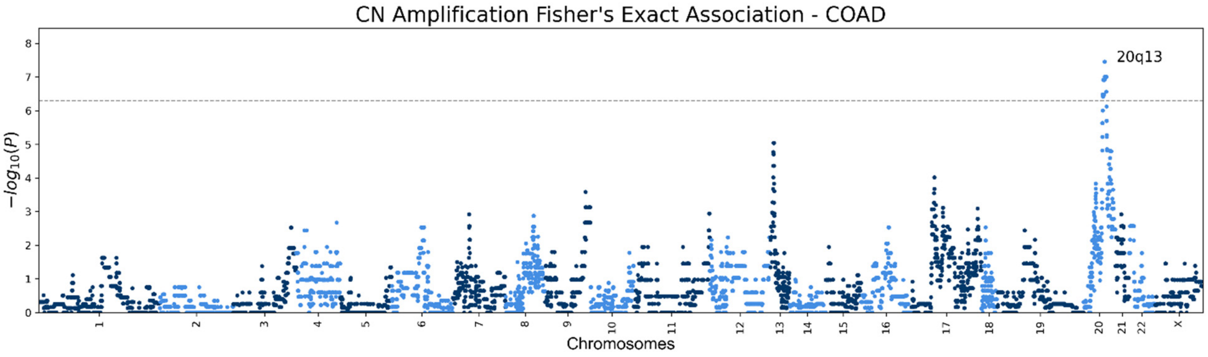

Figure S6. ESCA Manhattan plots

Genic Manhattan plot over Fisher's exact test p-values between ESCA chromoanagenesis samples and non-chromoanagenesis samples. **(a)** Manhattan CNA (deletion or amplification) plot. **(b)** Manhattan Deletion plot. **(c)** Manhattan Amplification plot.

a

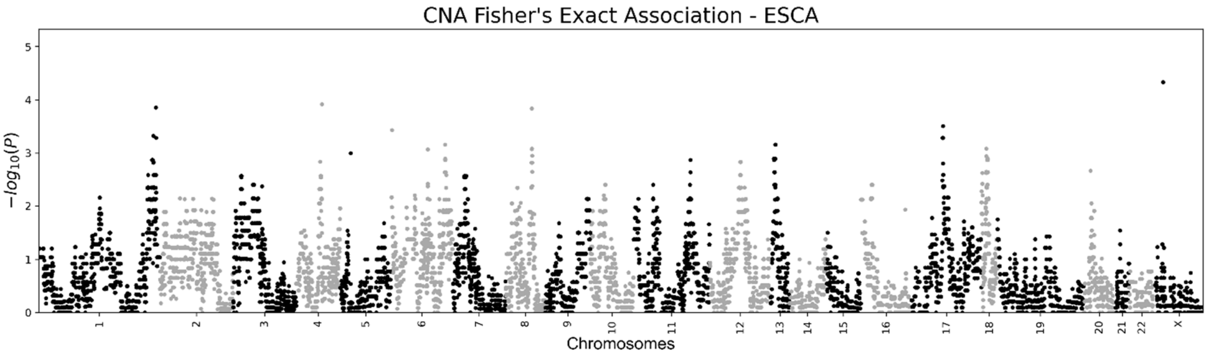

b

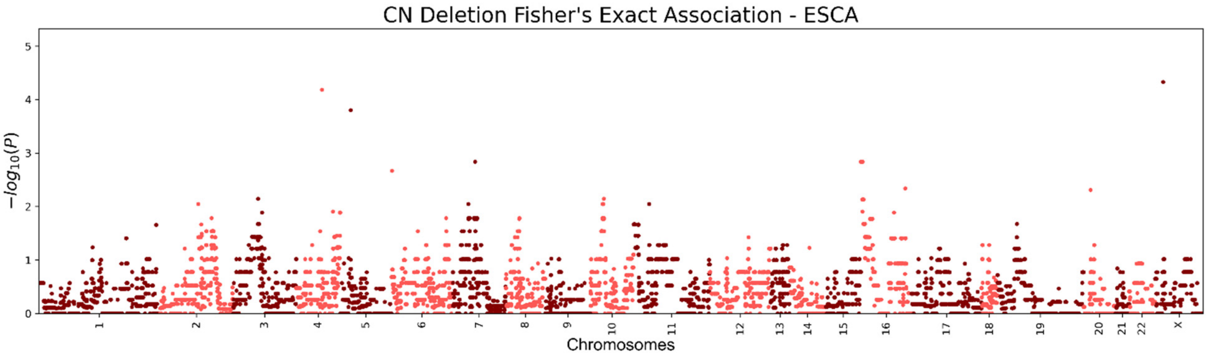

c

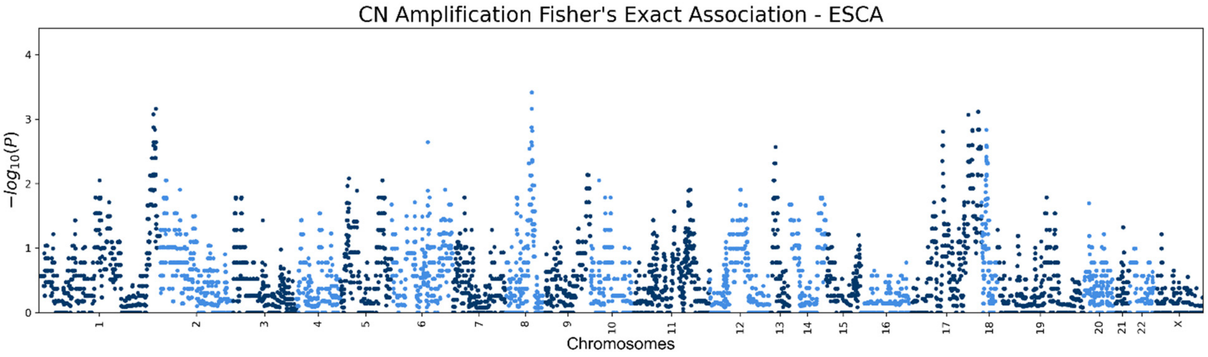

Figure S7. GBM Manhattan plots

Genic Manhattan plot over Fisher's exact test p-values between GBM chromoanagenesis samples and non-chromoanagenesis samples. **(a)** Manhattan CNA (deletion or amplification) plot. **(b)** Manhattan Deletion plot. **(c)** Manhattan Amplification plot.

a

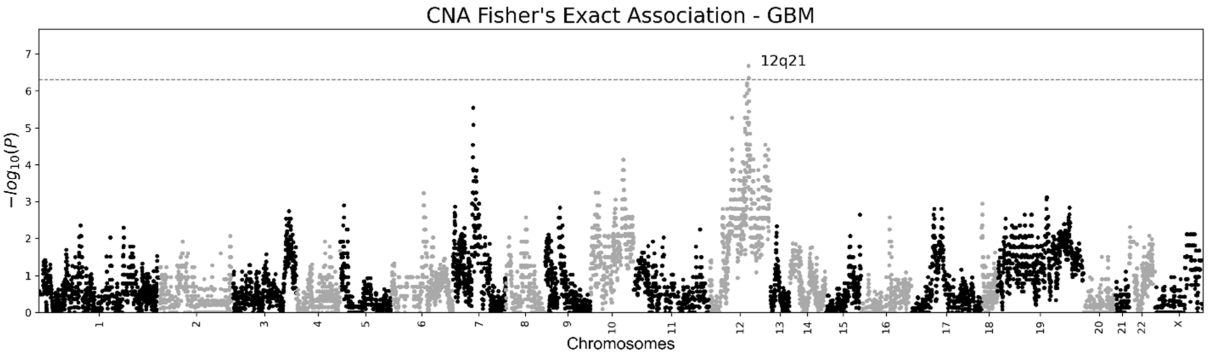

b

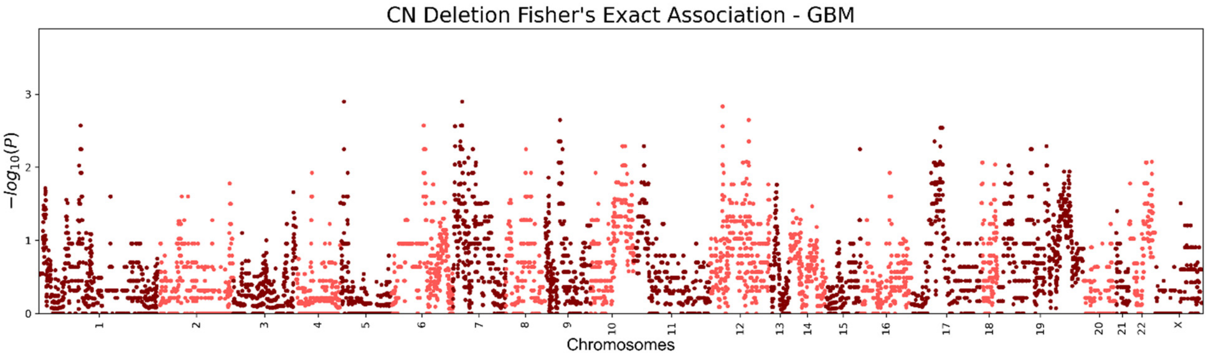

c

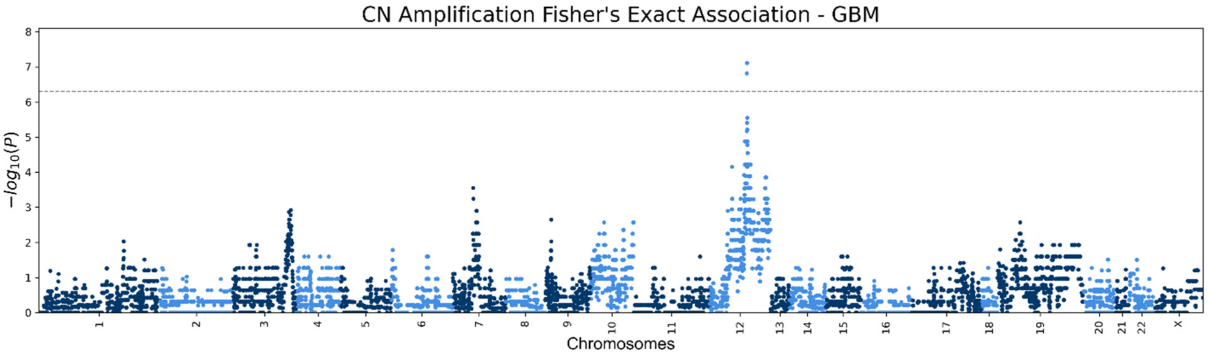

Figure S8. HNSC Manhattan plots

Genic Manhattan plot over Fisher's exact test p-values between HNSC chromoanagenesis samples and non-chromoanagenesis samples. **(a)** Manhattan CNA (deletion or amplification) plot. **(b)** Manhattan Deletion plot. **(c)** Manhattan Amplification plot.

a

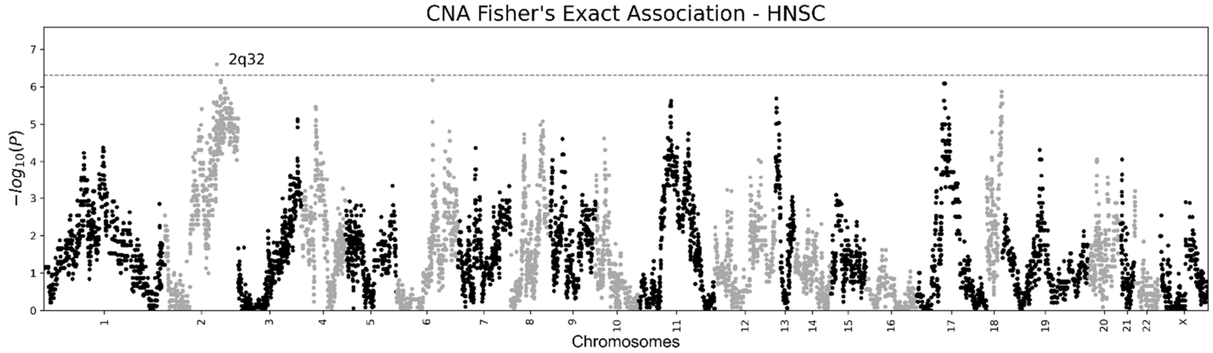

b

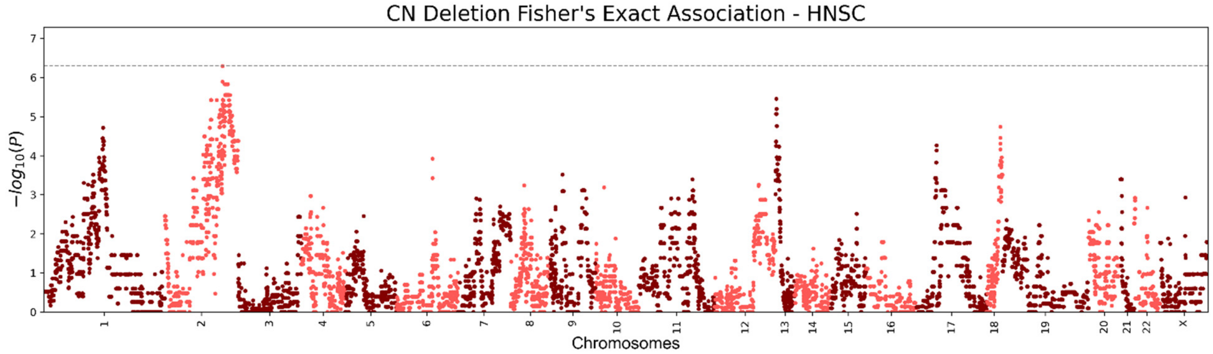

c

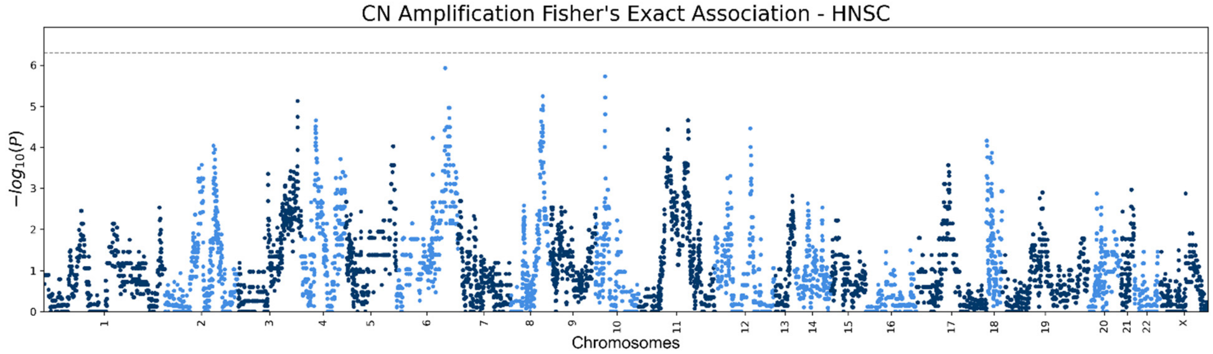

Figure S9. KIRC Manhattan plots

Genic Manhattan plot over Fisher's exact test p-values between KIRC chromoanagenesis samples and non-chromoanagenesis samples. **(a)** Manhattan CNA (deletion or amplification) plot. **(b)** Manhattan Deletion plot. **(c)** Manhattan Amplification plot.

a

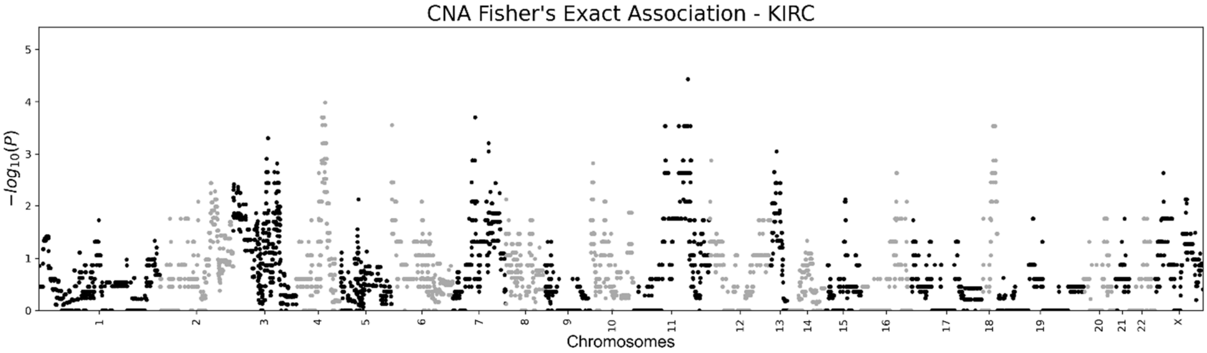

b

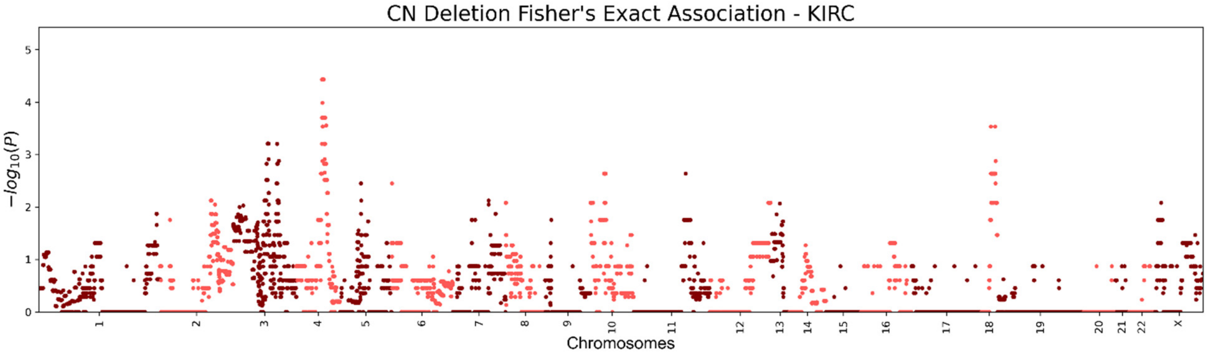

c

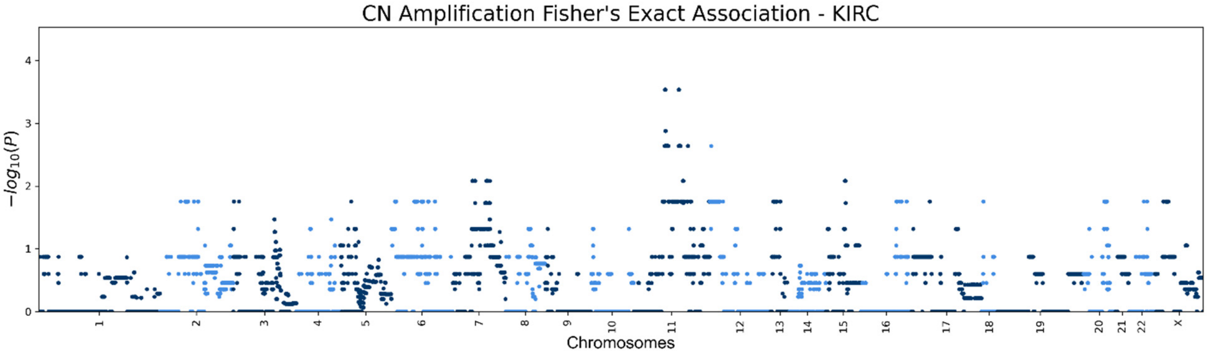

Figure S10. LIHC Manhattan plots

Genic Manhattan plot over Fisher's exact test p-values between LIHC chromoanagenesis samples and non-chromoanagenesis samples. **(a)** Manhattan CNA (deletion or amplification) plot. **(b)** Manhattan Deletion plot. **(c)** Manhattan Amplification plot.

a

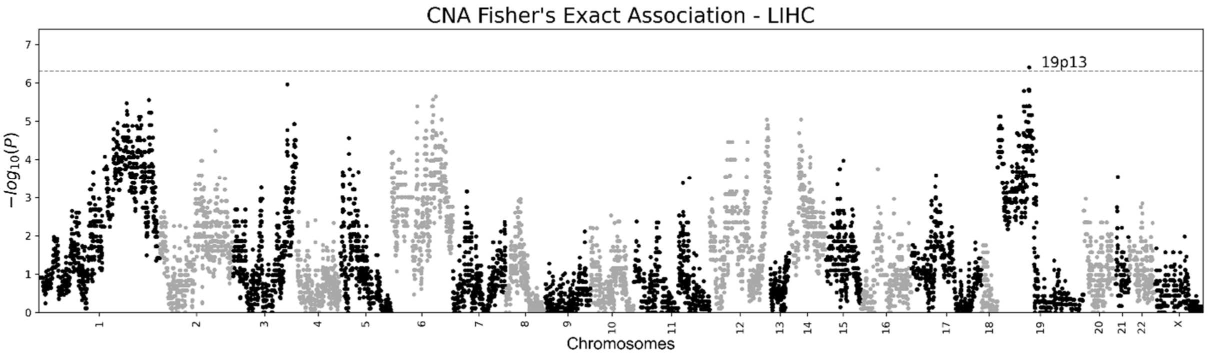

b

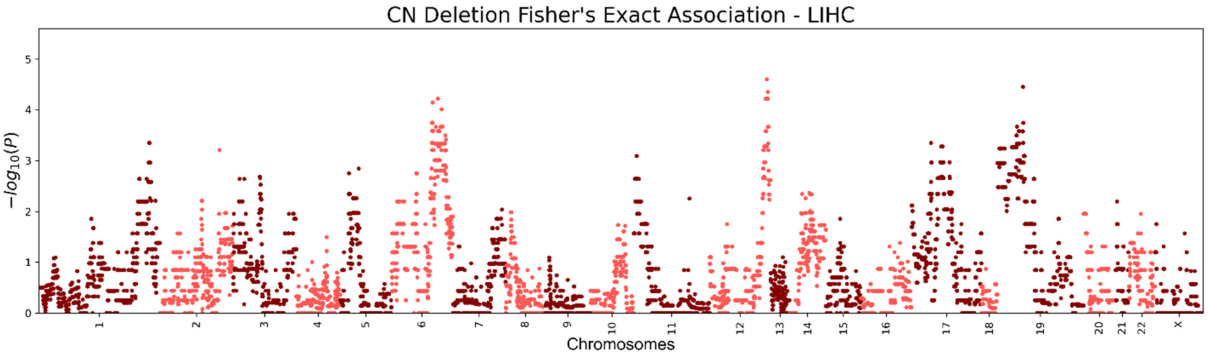

c

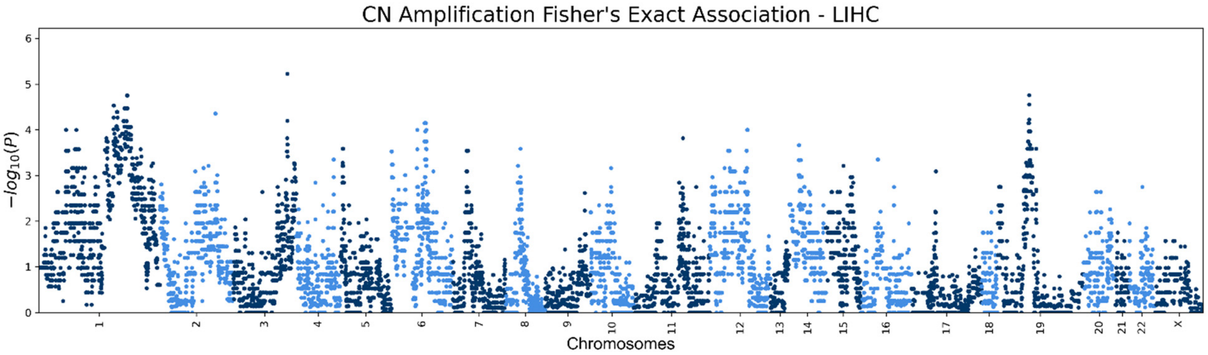

Figure S11. LUAD Manhattan plots

Genic Manhattan plot over Fisher's exact test p-values between LUAD chromoanagenesis samples and non-chromoanagenesis samples. **(a)** Manhattan CNA (deletion or amplification) plot. **(b)** Manhattan Deletion plot. **(c)** Manhattan Amplification plot.

a

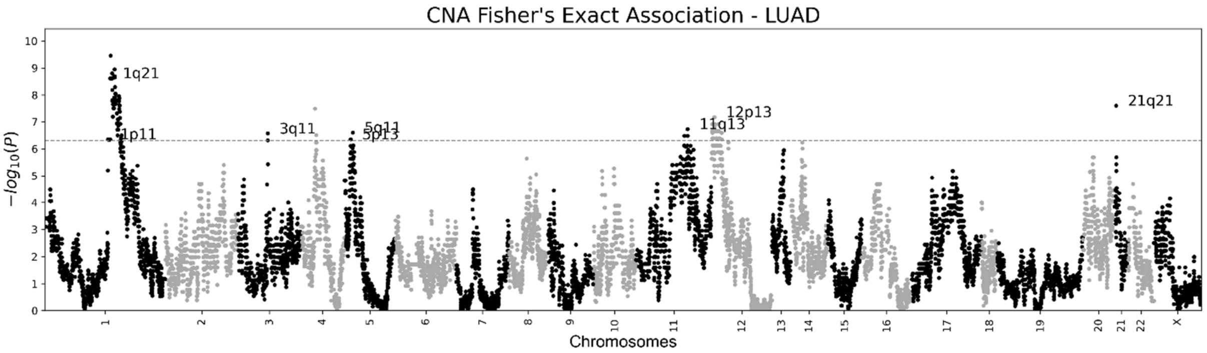

b

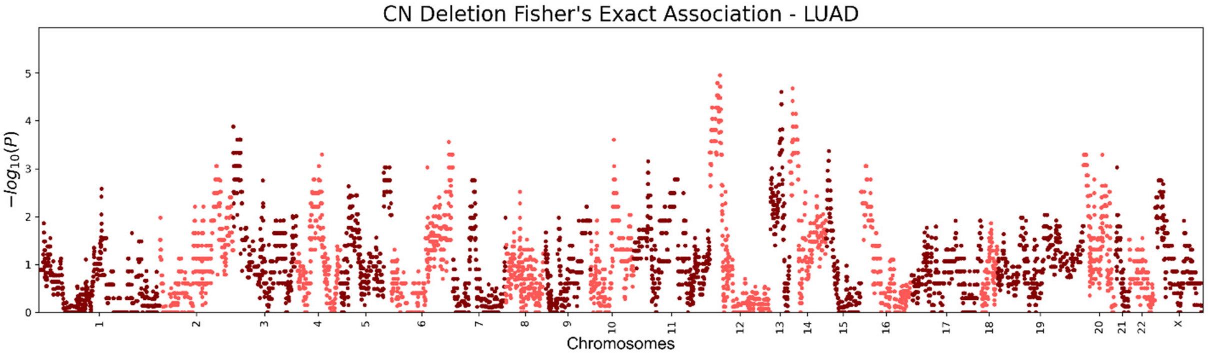

c

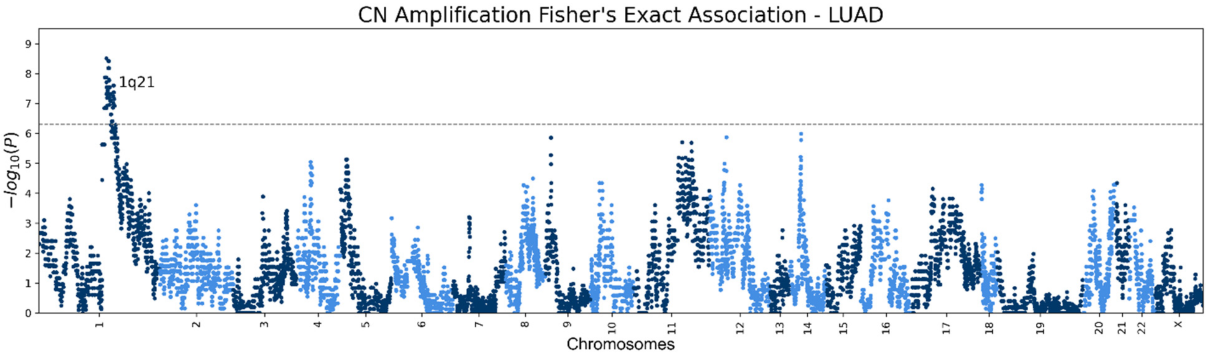

Figure S12. LUSC Manhattan plots

Genic Manhattan plot over Fisher's exact test p-values between LUSC chromoanagenesis samples and non-chromoanagenesis samples. **(a)** Manhattan CNA (deletion or amplification) plot. **(b)** Manhattan Deletion plot. **(c)** Manhattan Amplification plot.

a

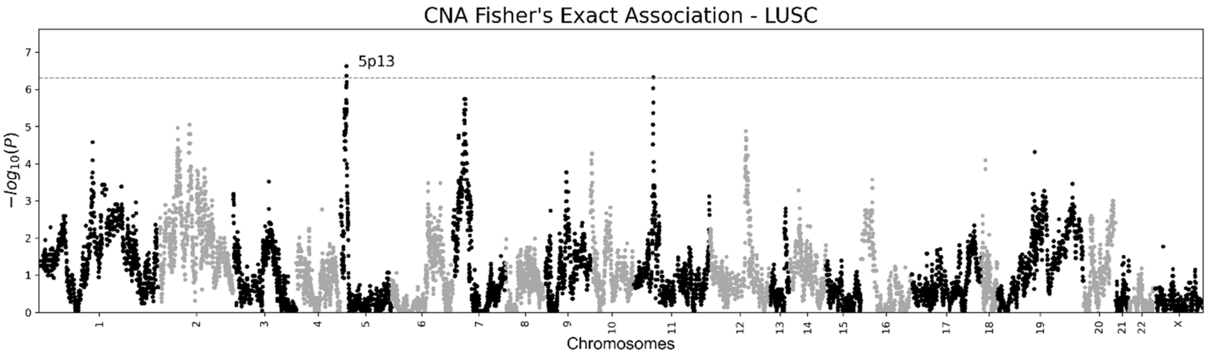

b

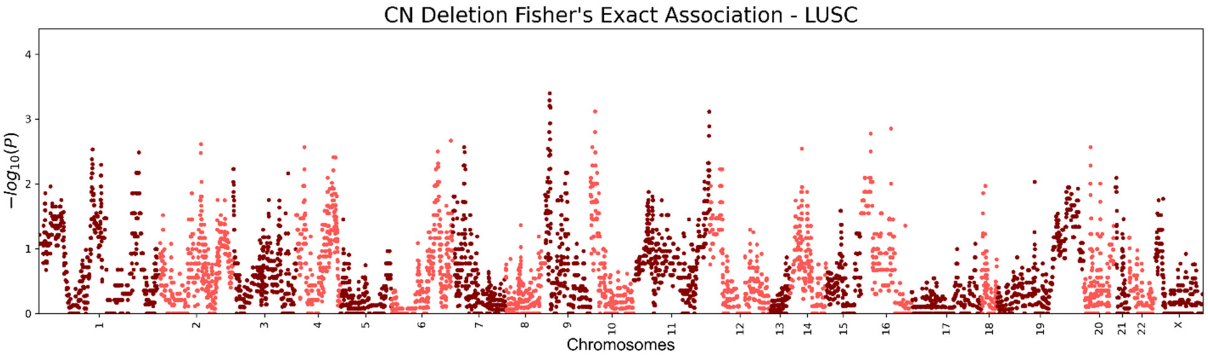

c

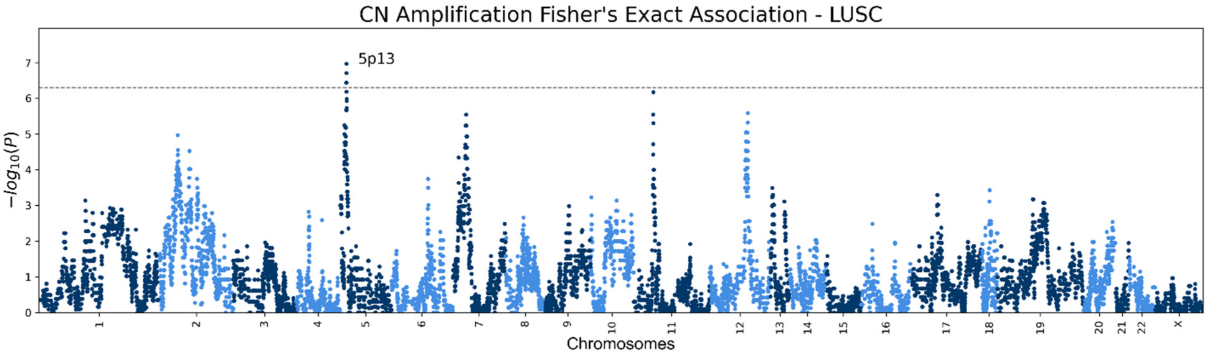

Figure S13. OV Manhattan plots

Genic Manhattan plot over Fisher's exact test p-values between OV chromoanagenesis samples and non-chromoanagenesis samples. **(a)** Manhattan CNA (deletion or amplification) plot. **(b)** Manhattan Deletion plot. **(c)** Manhattan Amplification plot.

a

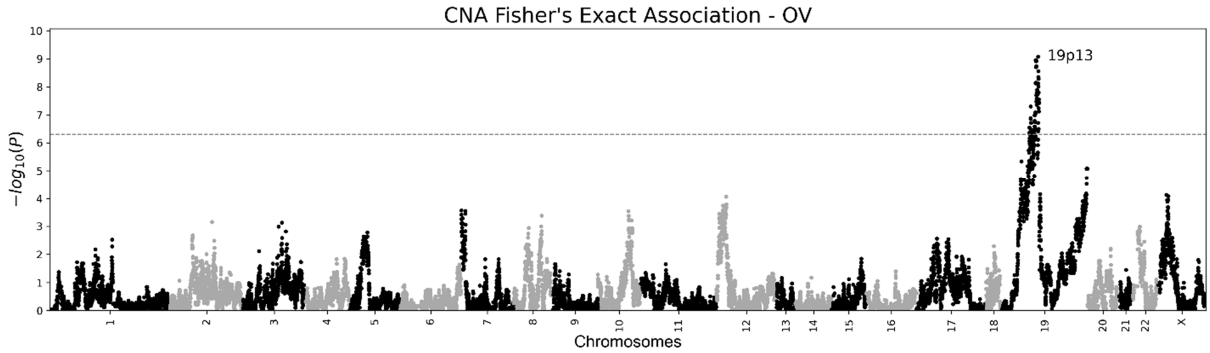

b

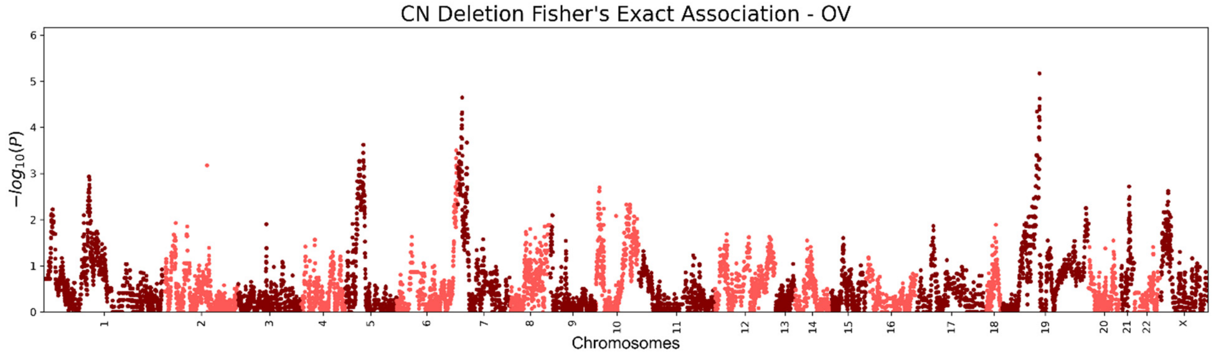

c

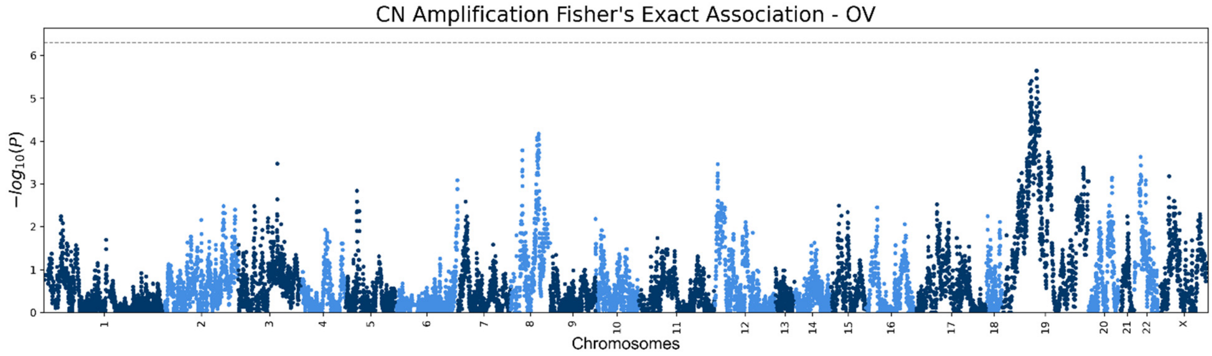

Figure S14. PAAD Manhattan plots

Genic Manhattan plot over Fisher's exact test p-values between PAAD chromoanagenesis samples and non-chromoanagenesis samples. **(a)** Manhattan CNA (deletion or amplification) plot. **(b)** Manhattan Deletion plot. **(c)** Manhattan Amplification plot.

a

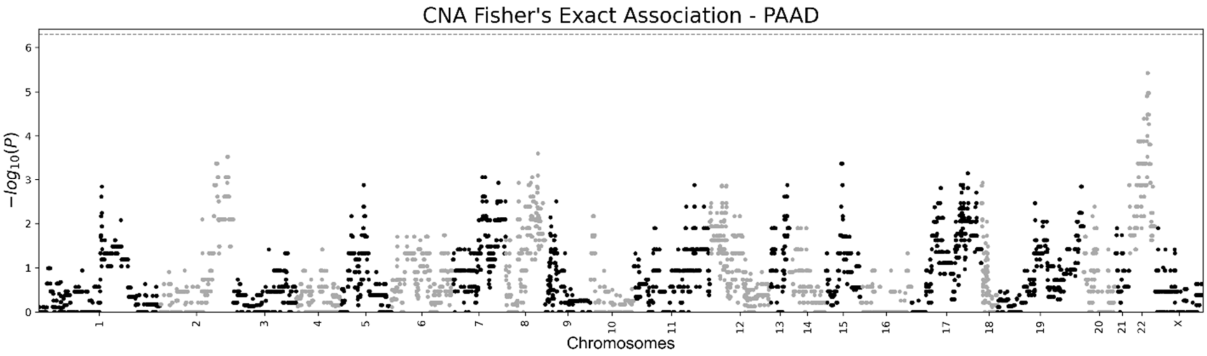

b

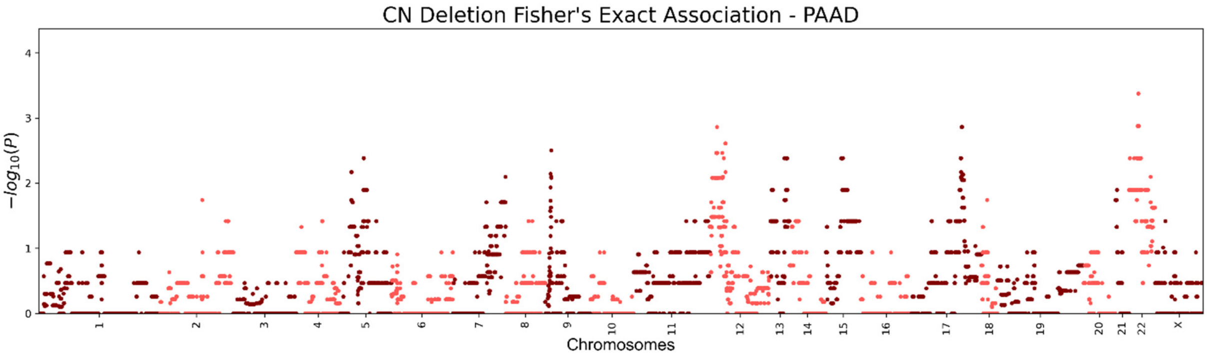

c

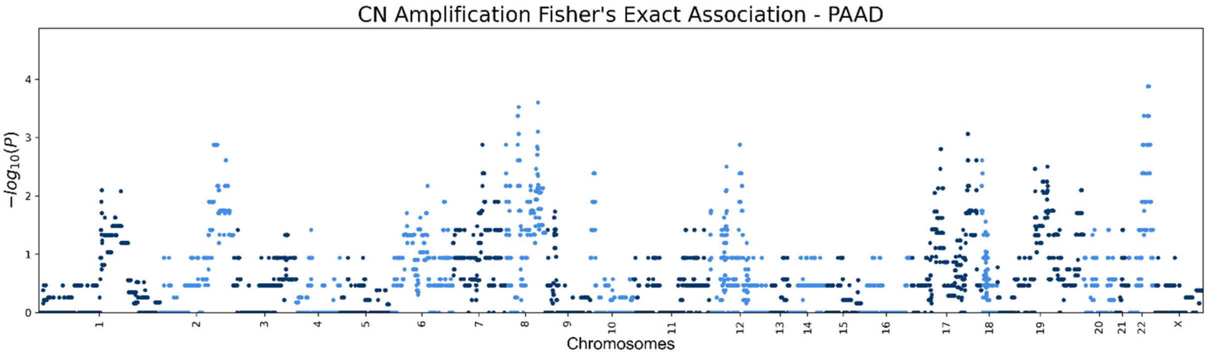

Figure S15. PRAD Manhattan plots

Genic Manhattan plot over Fisher's exact test p-values between PRAD chromoanagenesis samples and non-chromoanagenesis samples. **(a)** Manhattan CNA (deletion or amplification) plot. **(b)** Manhattan Deletion plot. **(c)** Manhattan Amplification plot.

a

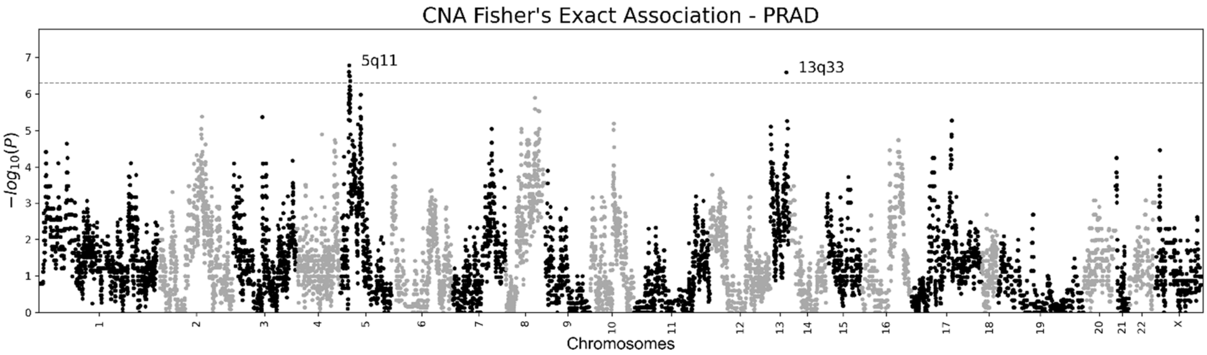

b

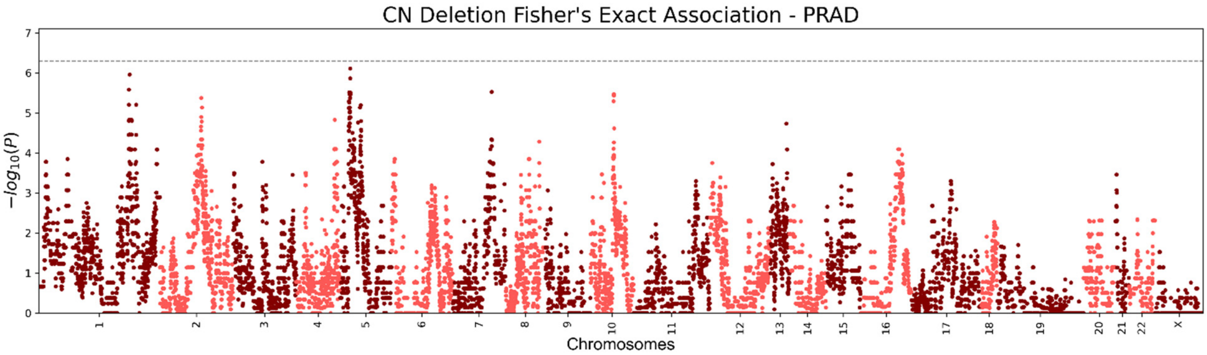

c

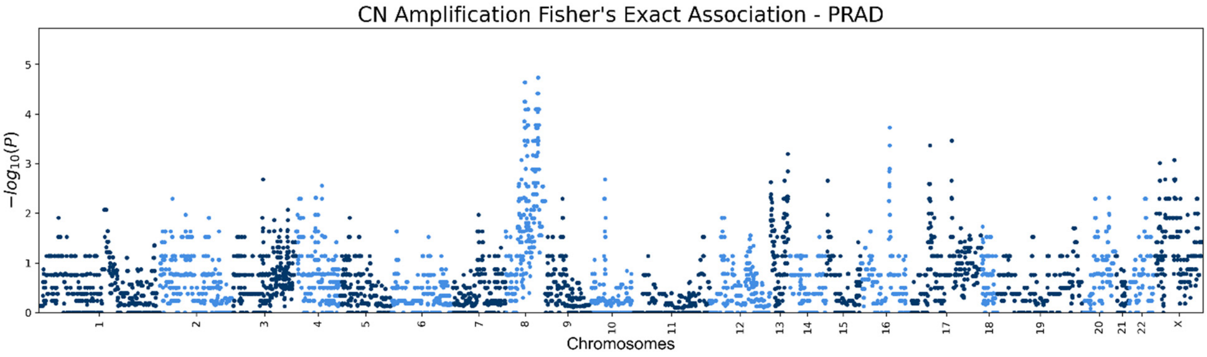

Figure S16. READ Manhattan plots

Genic Manhattan plot over Fisher's exact test p-values between READ chromoanagenesis samples and non-chromoanagenesis samples. **(a)** Manhattan CNA (deletion or amplification) plot. **(b)** Manhattan Deletion plot. **(c)** Manhattan Amplification plot.

a

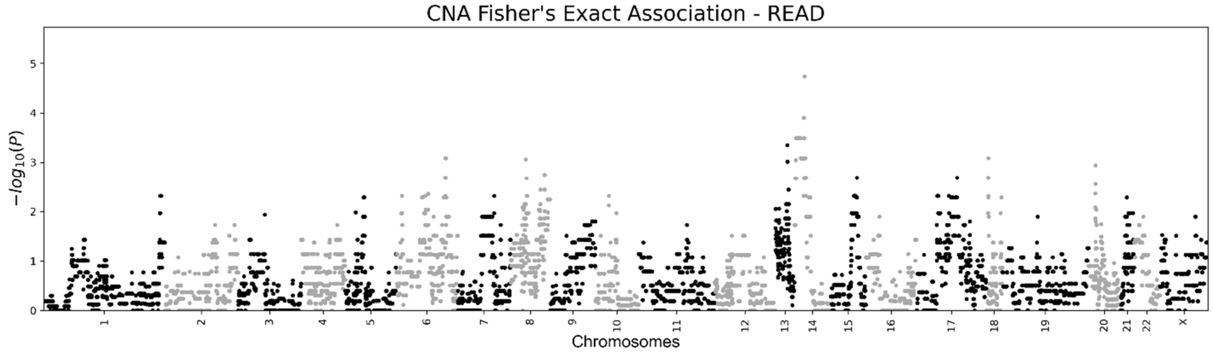

b

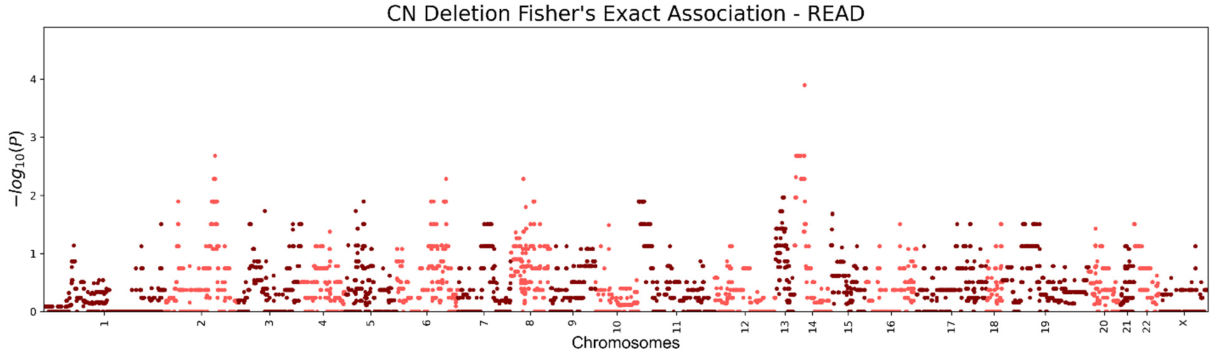

c

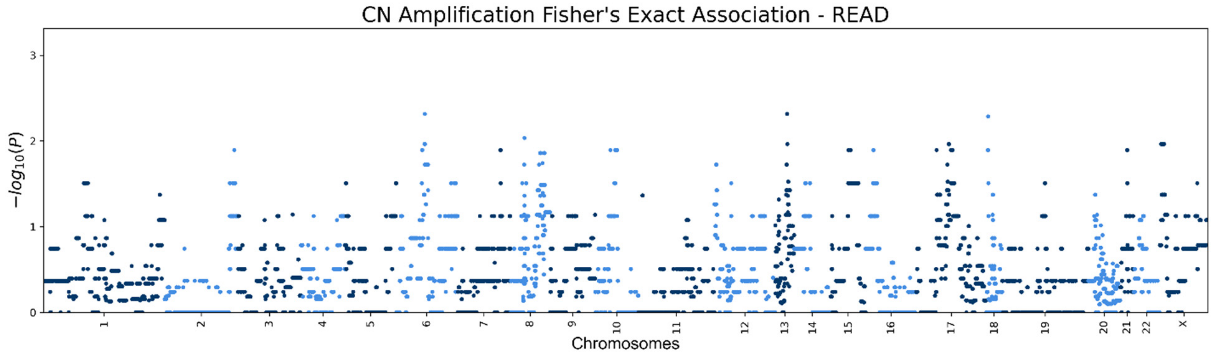

Figure S17. SARC Manhattan plots

Genic Manhattan plot over Fisher's exact test p-values between SARC chromoanagenesis samples and non-chromoanagenesis samples. **(a)** Manhattan CNA (deletion or amplification) plot. **(b)** Manhattan Deletion plot. **(c)** Manhattan Amplification plot.

a

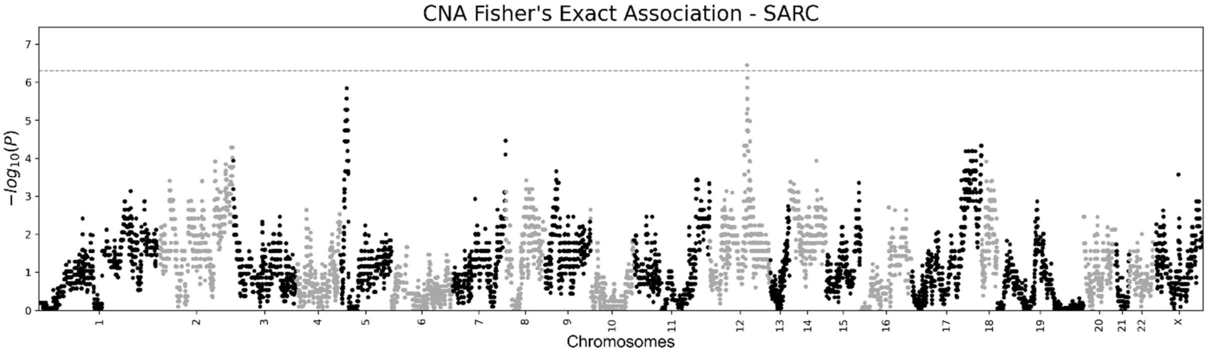

b

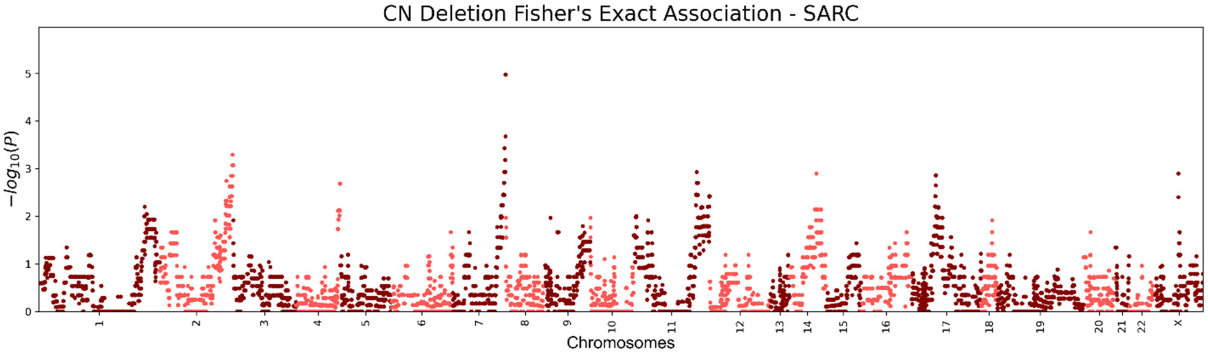

c

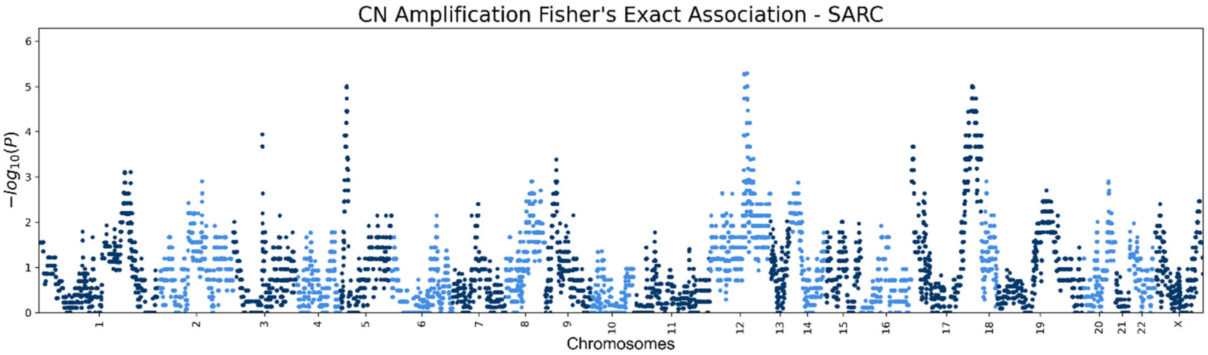

Figure S18. SKCM Manhattan plots

Genic Manhattan plot over Fisher's exact test p-values between SKCM chromoanagenesis samples and non-chromoanagenesis samples. **(a)** Manhattan CNA (deletion or amplification) plot. **(b)** Manhattan Deletion plot. **(c)** Manhattan Amplification plot.

a

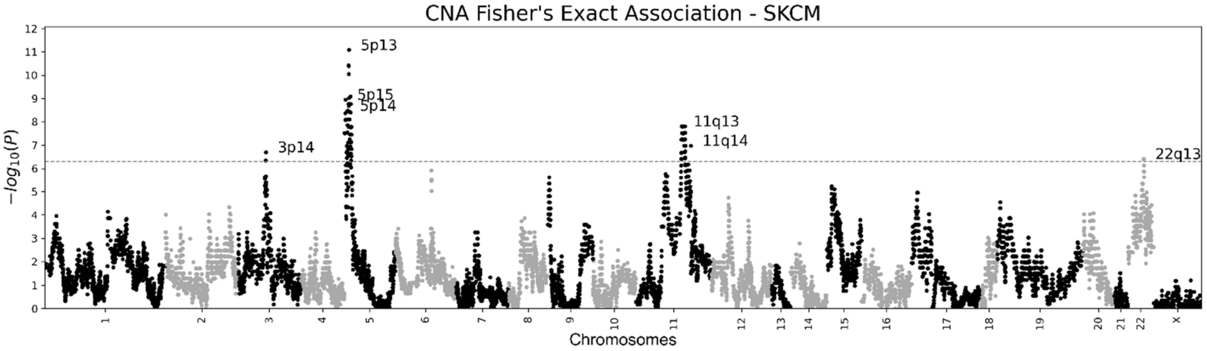

b

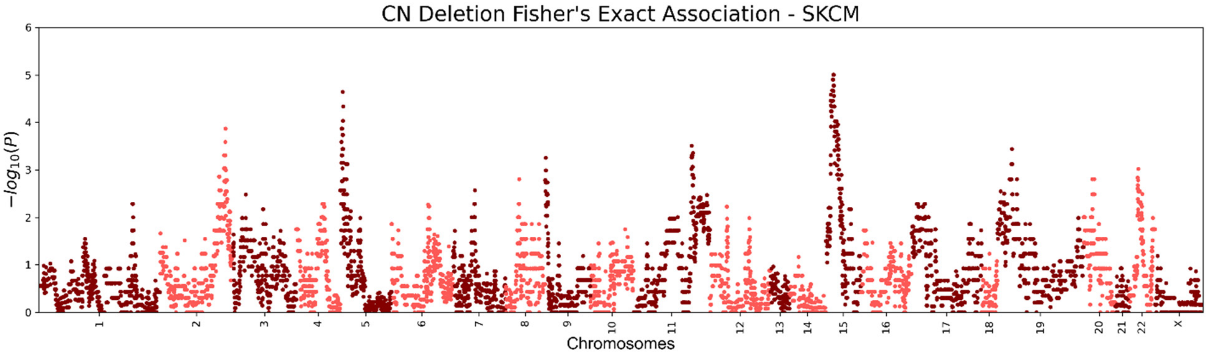

c

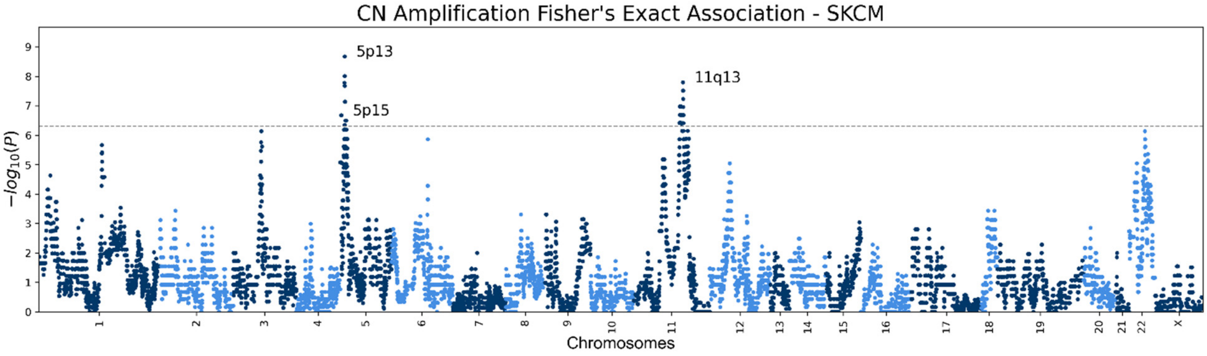

Figure S19. STAD Manhattan plots

Genic Manhattan plot over Fisher's exact test p-values between STAD chromoanagenesis samples and non-chromoanagenesis samples. **(a)** Manhattan CNA (deletion or amplification) plot. **(b)** Manhattan Deletion plot. **(c)** Manhattan Amplification plot.

a

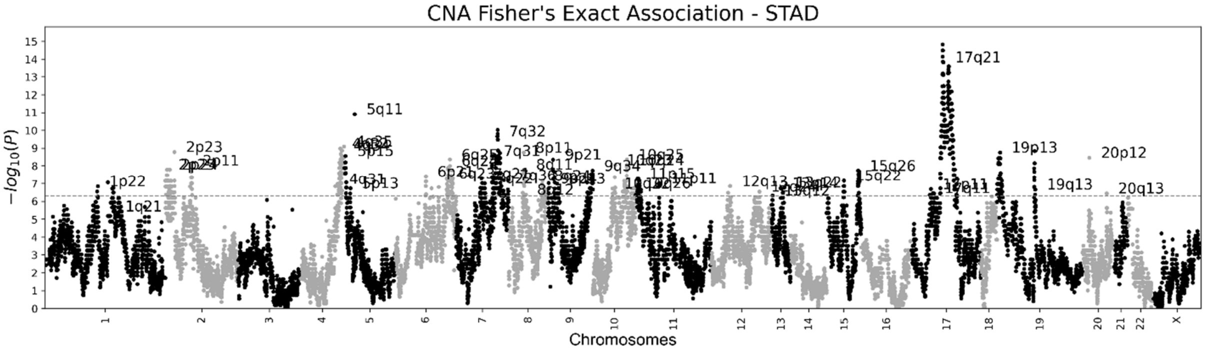

b

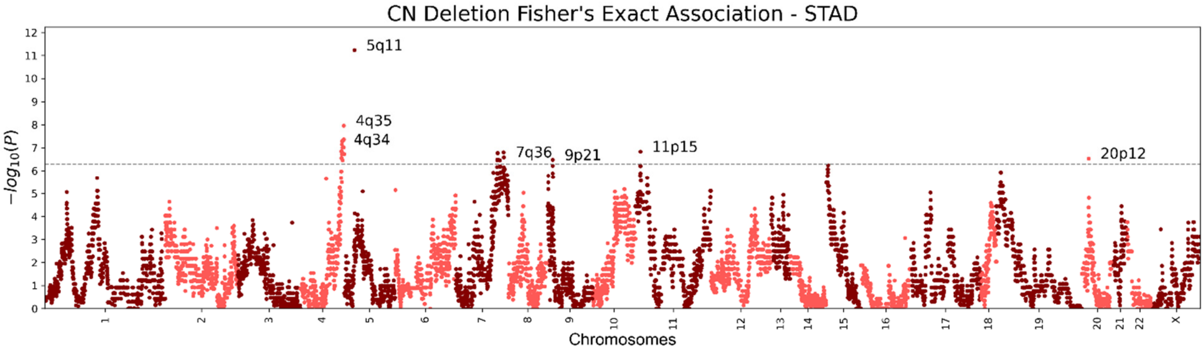

c

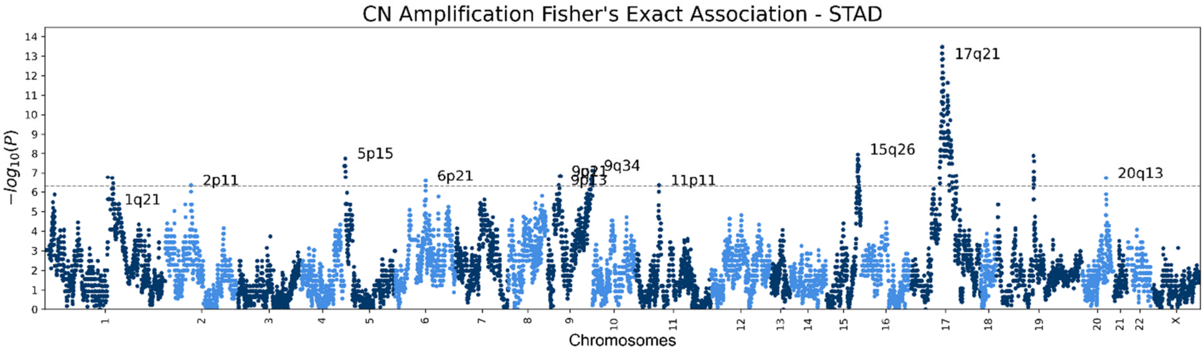

Figure S20. UCEC Manhattan plots

Genic Manhattan plot over Fisher's exact test p-values between UCEC chromoanagenesis samples and non-chromoanagenesis samples. **(a)** Manhattan CNA (deletion or amplification) plot. **(b)** Manhattan Deletion plot. **(c)** Manhattan Amplification plot.

a

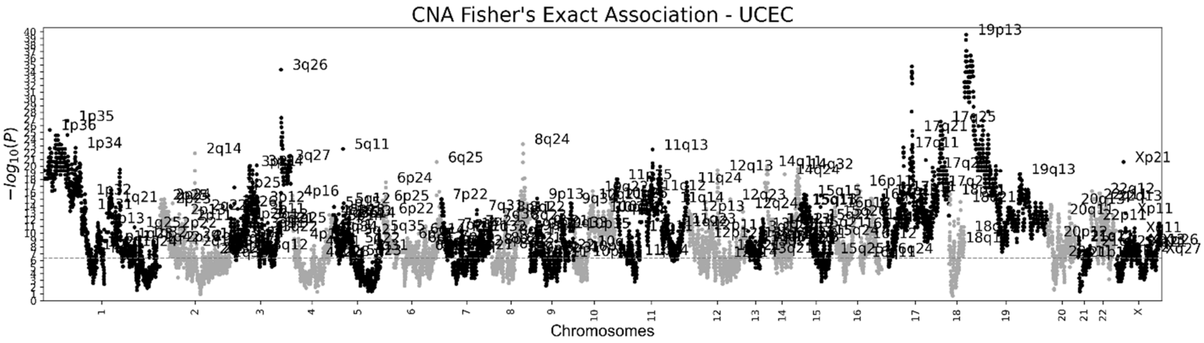

b

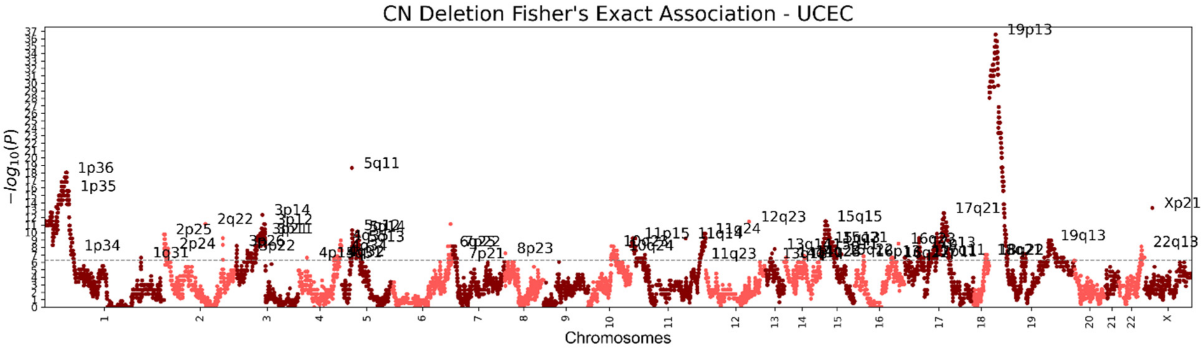

c

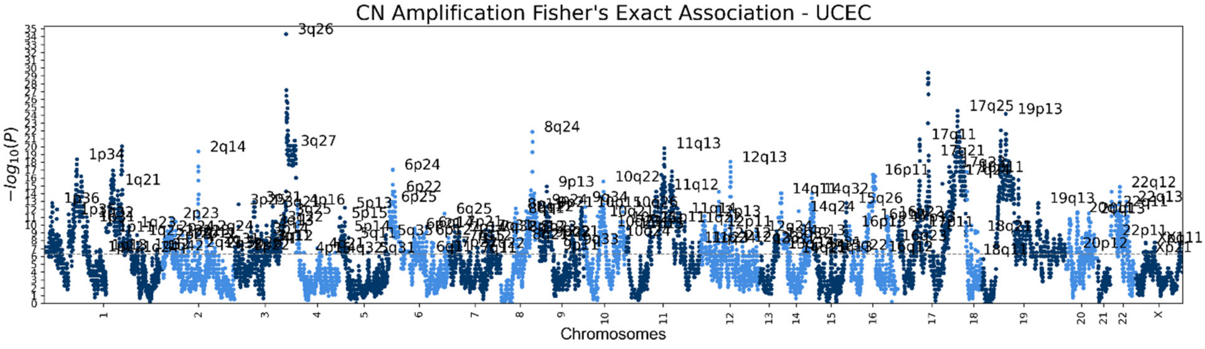

Figure S21. (a) Kaplan-Meier Overall survival rate estimate for chromoanagenesis in BLCA. **(b)** Cox regression hazard ratio, for chromoanagenesis, age and gender in BLCA patients.

a

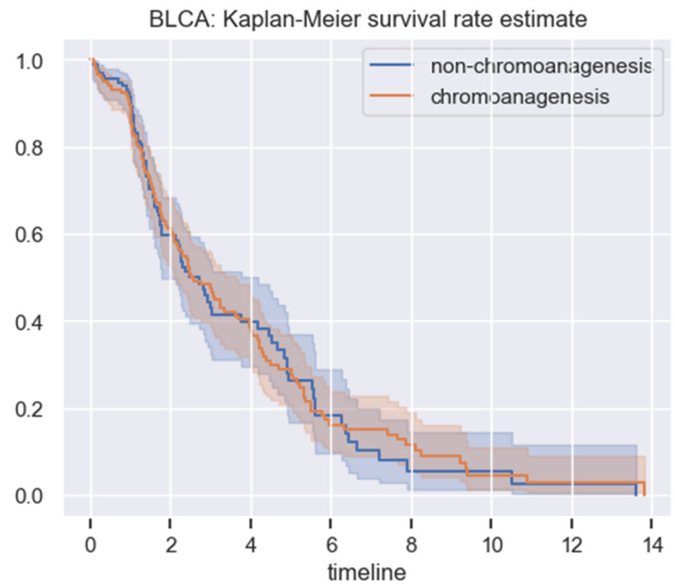

**chromoanagenesis**								
At risk	205	79	39	15	10	3	2	0
Censored	0	63	75	78	79	81	81	81
Events	0	63	91	112	116	121	122	124
**non-chromoanagenesis**								
At risk	158	52	25	9	2	2	1	0
Censored	0	60	72	76	77	77	77	77
Events	0	46	61	73	79	79	80	81

b

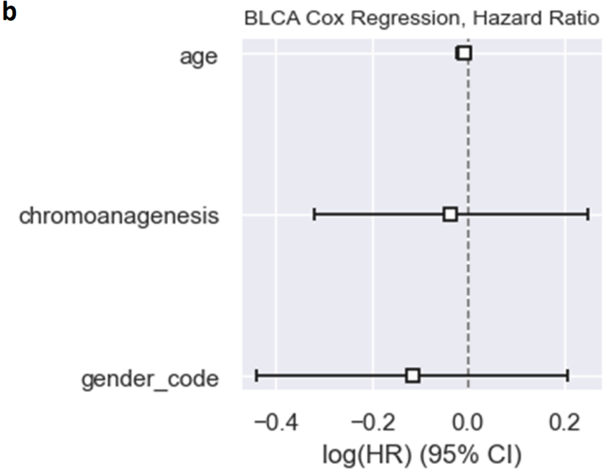

Figure S22. **(a)** Kaplan-Meier Overall survival rate estimate for chromoanagenesis in BRCA. **(b)** Cox regression hazard ratio, for chromoanagenesis, age and gender in BRCA patients.

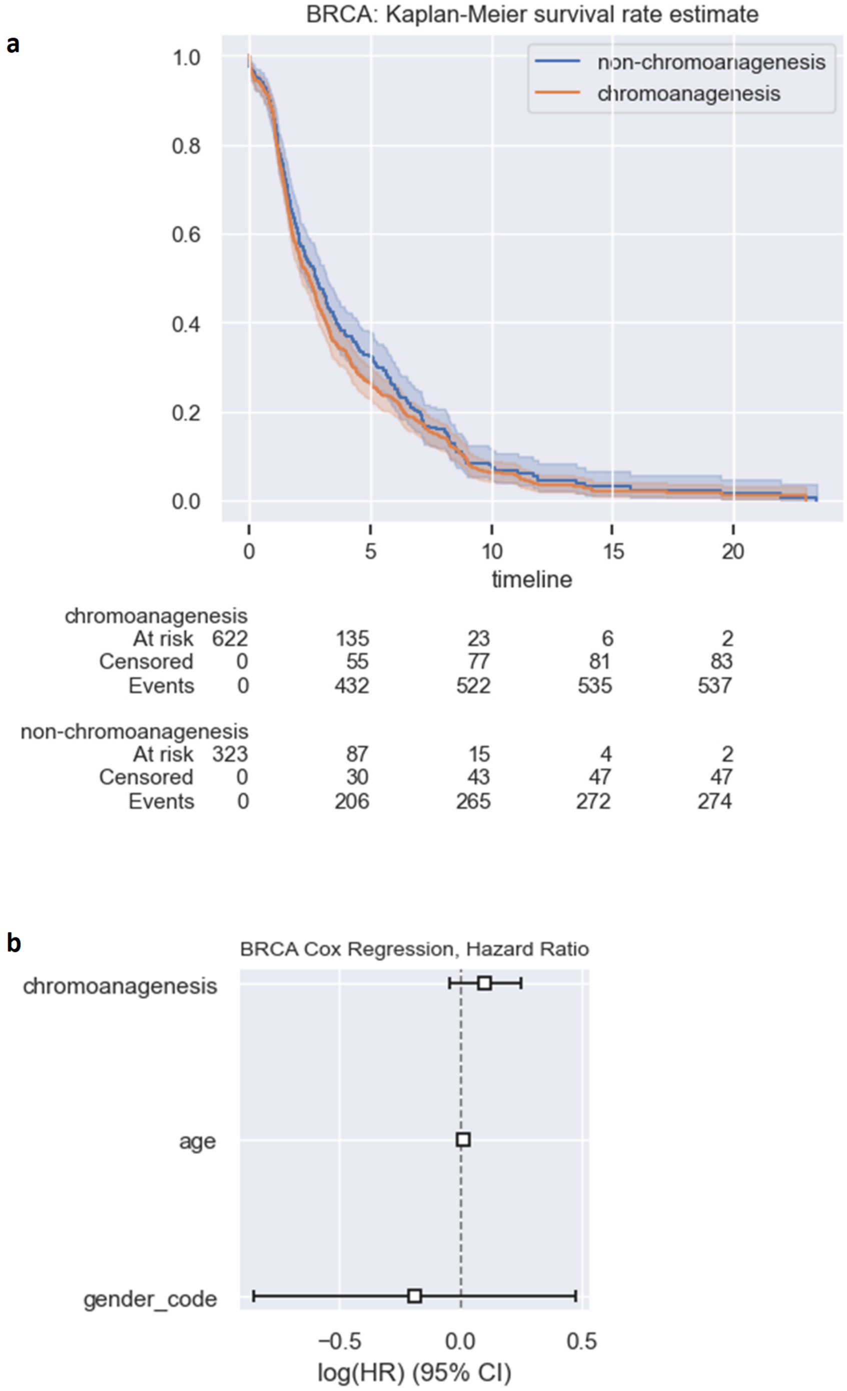

Figure S23. Kaplan-Meier Overall survival rate estimate for chromoanagenesis in CESC.

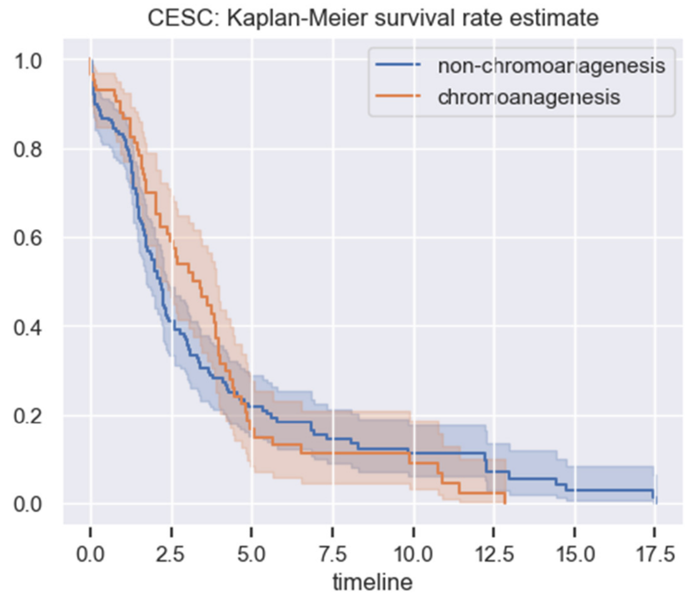

**chromoanagenesis**								
At risk	84	36	9	6	4	1	0	0
Censored	0	18	22	22	23	23	23	23
Events	0	30	53	56	57	60	61	61
**non-chromoanagenesis**								
At risk	175	59	27	15	9	5	2	1
Censored	0	23	29	33	36	37	37	37
Events	0	93	119	127	130	133	136	137

Figure S24. **(a)** Kaplan-Meier Overall survival rate estimate for chromoanagenesis in COAD. **(b)** Cox regression hazard ratio, for chromoanagenesis, age and gender in COAD patients.

a

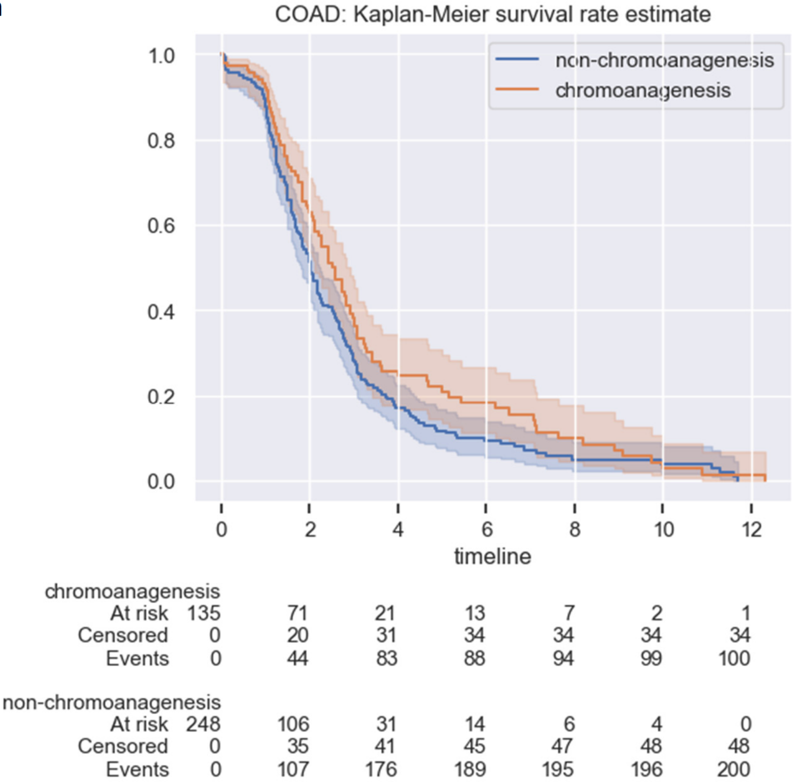

b

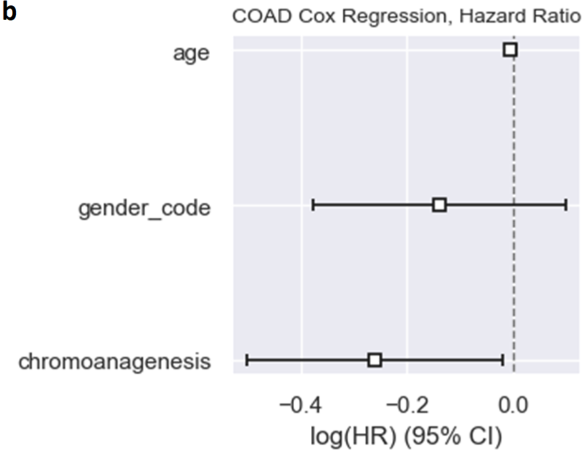

Figure S25. **(a)** Kaplan-Meier Overall survival rate estimate for chromoanagenesis in ESCA. **(b)** Cox regression hazard ratio, for chromoanagenesis, age and gender in ESCA patients.

a

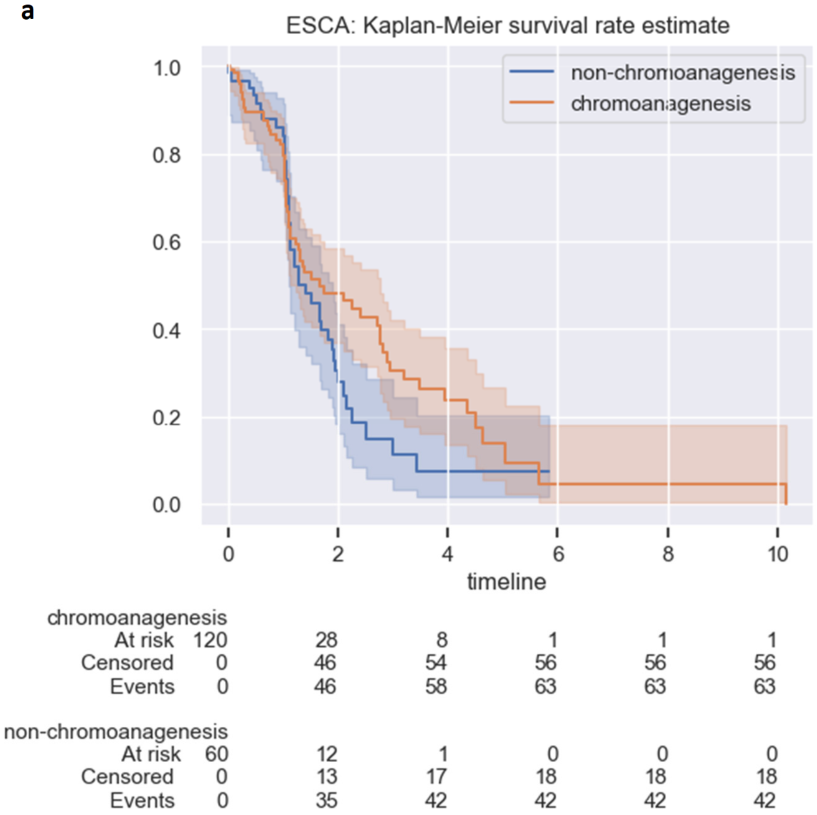

b

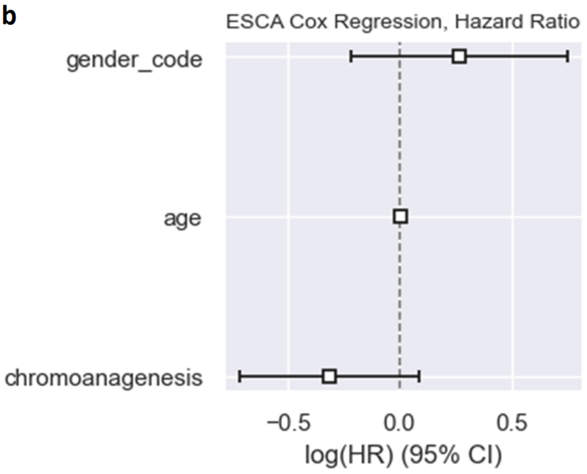

Figure S26. (a) Kaplan-Meier Overall survival rate estimate for chromoanagenesis in GBM. **(b)** Cox regression hazard ratio, for chromoanagenesis, age and gender in GBM patients.

a

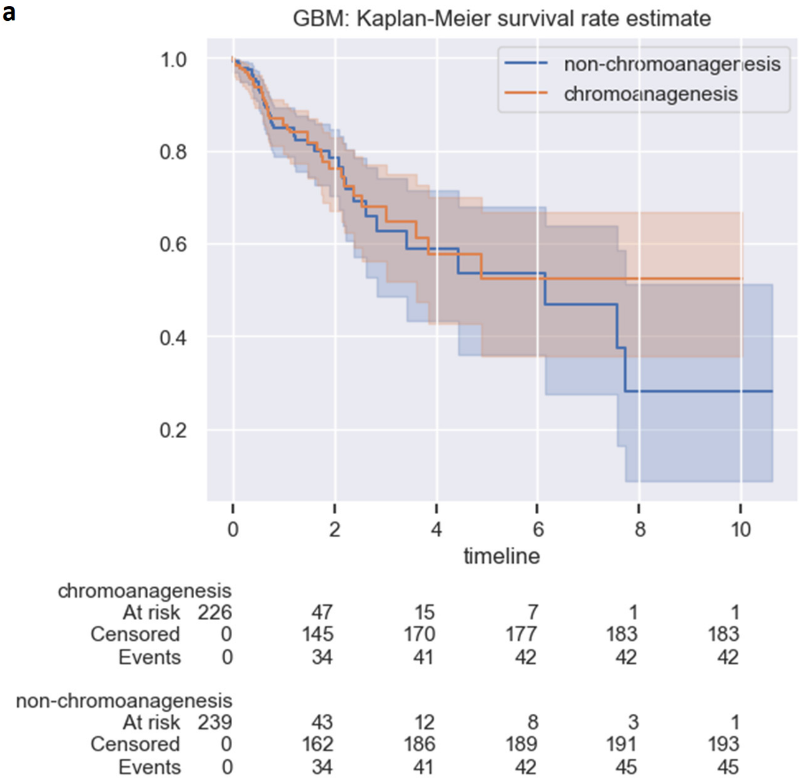

b

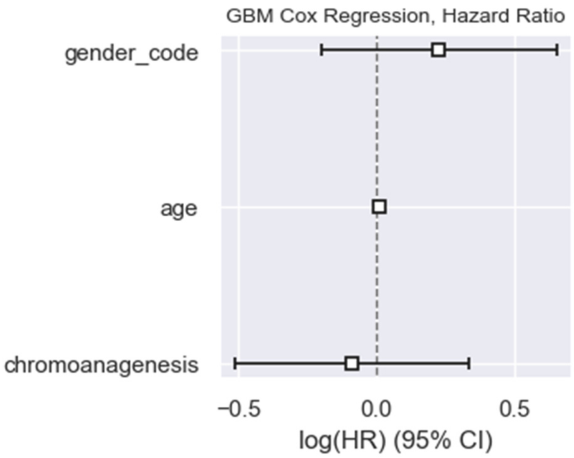

Figure S27. **(a)** Kaplan-Meier Overall survival rate estimate for chromoanagenesis in HNSC. **(b)** Cox regression hazard ratio, for chromoanagenesis, age and gender in HNSC patients.

a

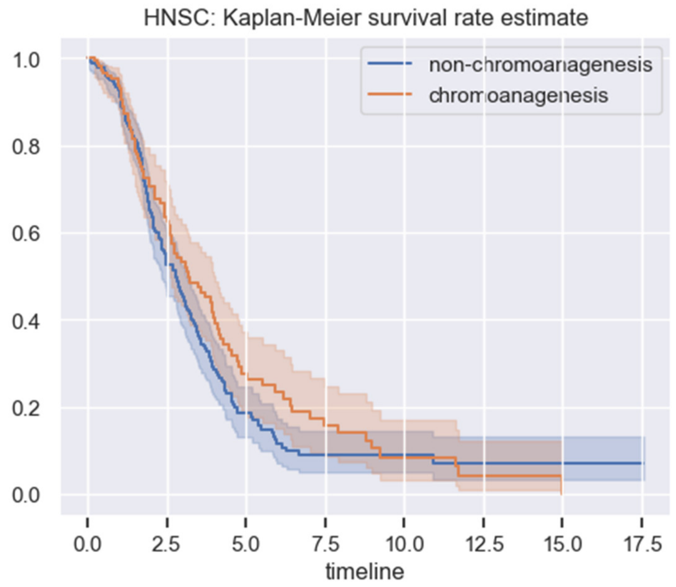

**chromoanagenesis**								
At risk	157	63	20	10	4	2	1	0
Censored	0	51	63	65	67	67	68	68
Events	0	43	74	82	86	88	88	89
**non-chromoanagenesis**								
At risk	311	98	27	7	5	4	1	1
Censored	0	108	121	129	131	131	134	134
Events	0	105	163	175	175	176	176	176

b

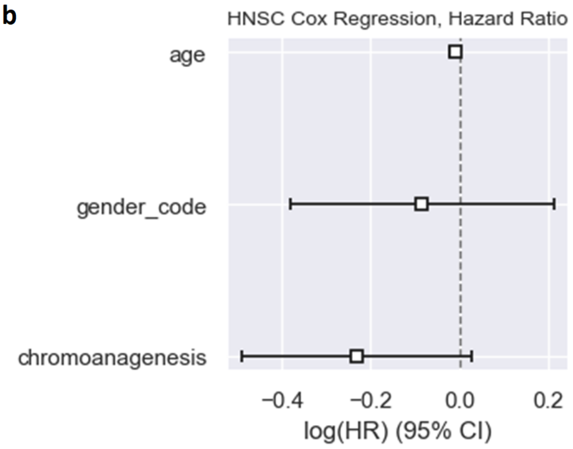

Figure S28. **(a)** Kaplan-Meier Overall survival rate estimate for chromoanagenesis in KIRC. **(b)** Cox regression hazard ratio, for chromoanagenesis, age and gender in KIRC patients.

a

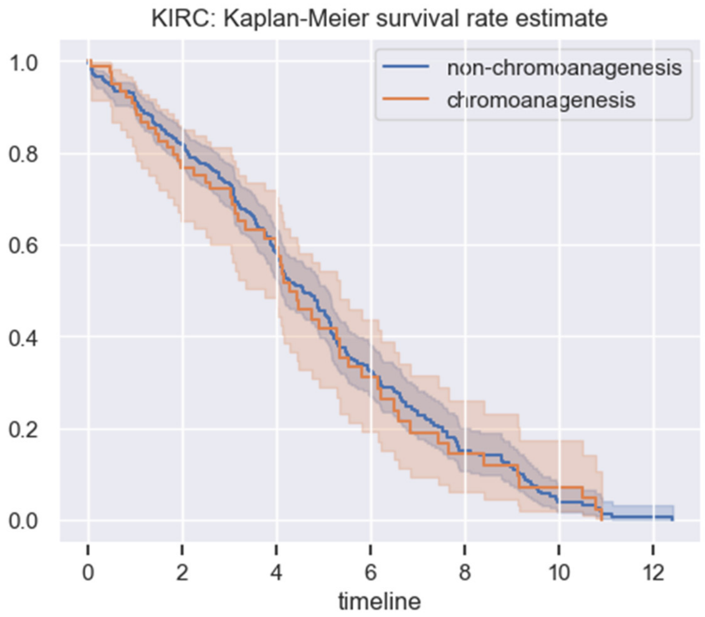

**chromoanagenesis**							
At risk	80	52	32	13	6	3	0
Censored	0	11	22	26	26	26	26
Events	0	17	26	41	48	51	54
**non-chromoanagenesis**							
At risk	395	267	161	75	29	6	1
Censored	0	62	97	118	128	130	130
Events	0	66	137	202	238	259	264

b

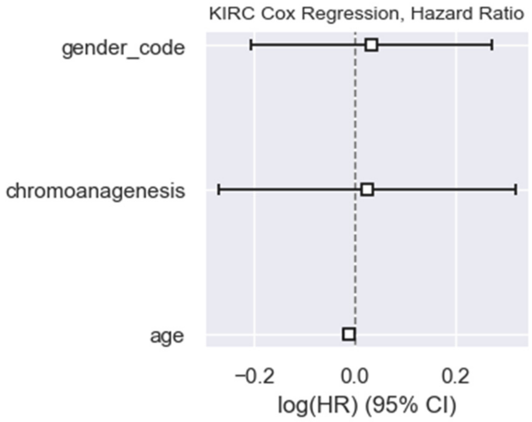

Figure S29. **(a)** Kaplan-Meier Overall survival rate estimate for chromoanagenesis in LGG. **(b)** Cox regression hazard ratio, for chromoanagenesis, age and gender in LGG patients.

a

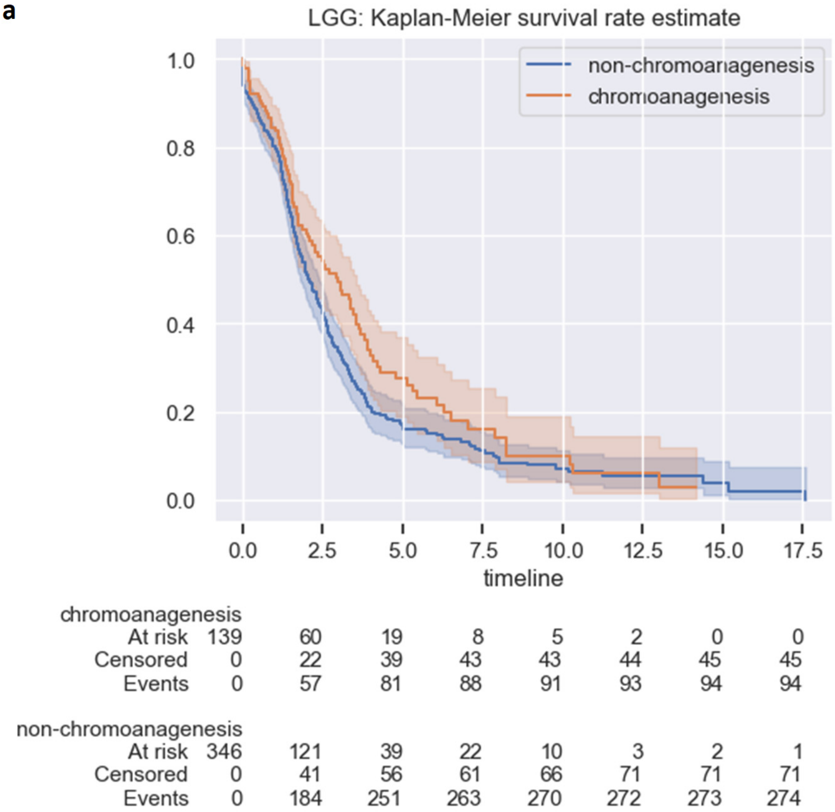

b

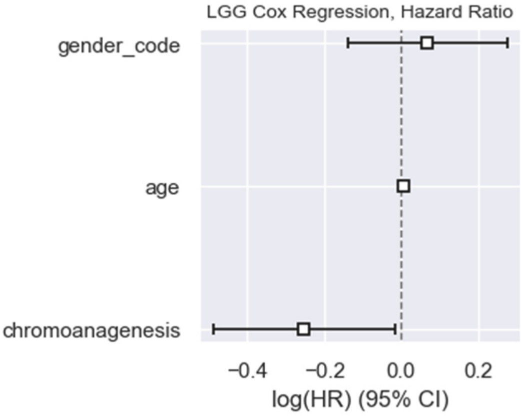

Figure S30. **(a)** Kaplan-Meier Overall survival rate estimate for chromoanagenesis in LIHC. **(b)** Cox regression hazard ratio, for chromoanagenesis, age and gender in LIHC patients.

a

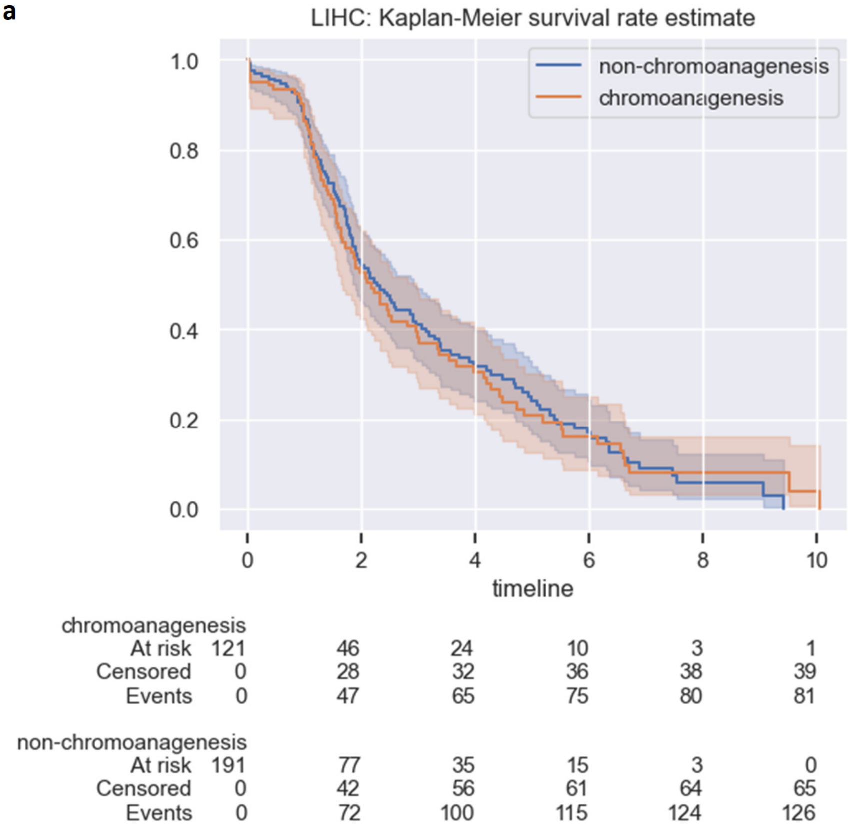

b

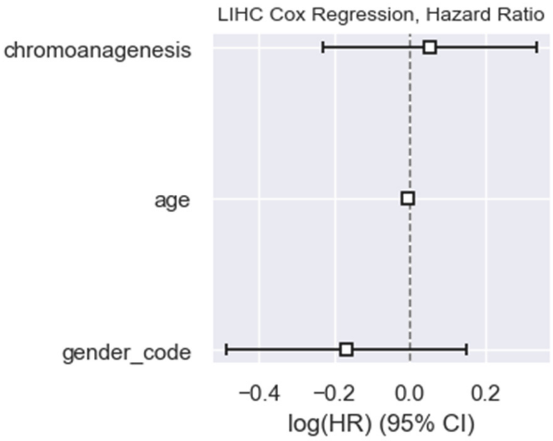

Figure S31. (a) Kaplan-Meier Overall survival rate estimate for chromoanagenesis in LUAD. **(b)** Cox regression hazard ratio, for chromoanagenesis, age and gender in LUAD patients.

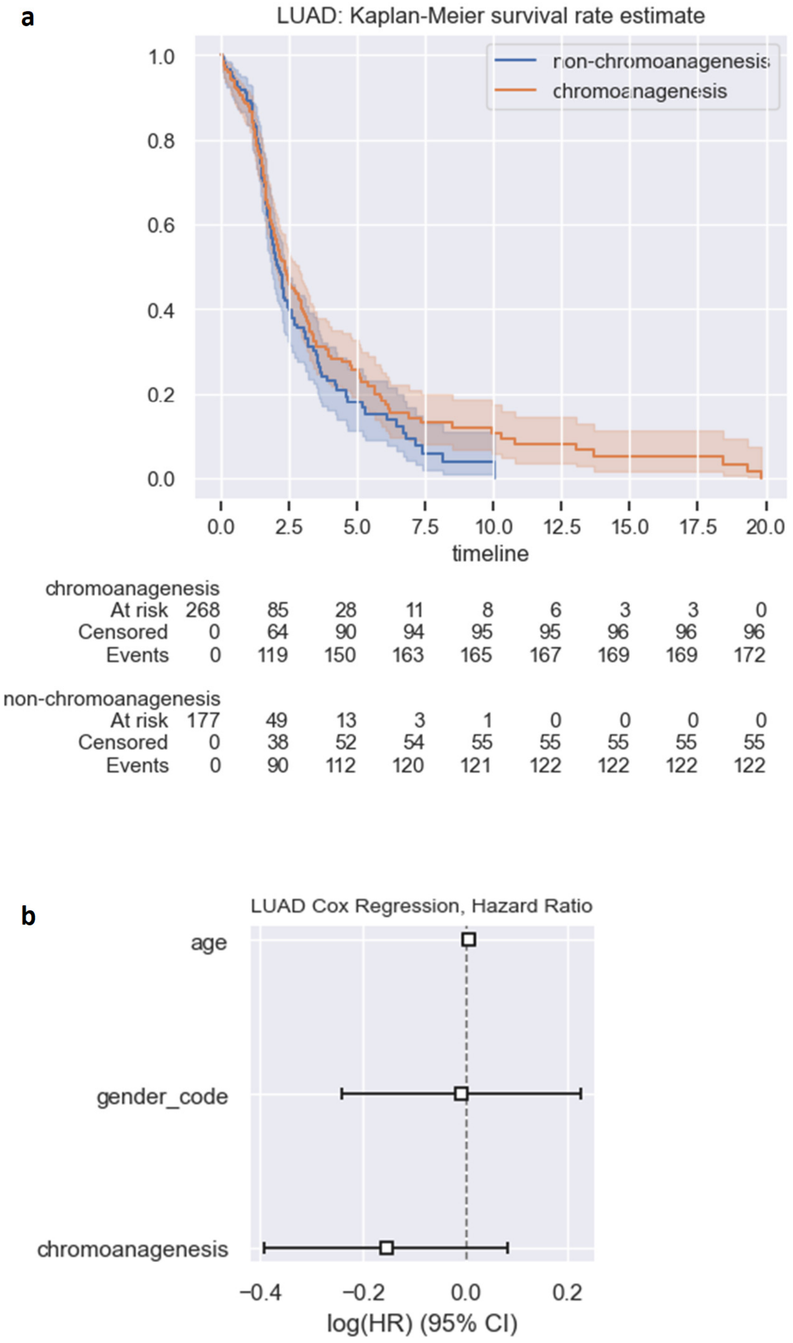

Figure S32. **(a)** Kaplan-Meier Overall survival rate estimate for chromoanagenesis in LUSC. **(b)** Cox regression hazard ratio, for chromoanagenesis, age and gender in LUSC patients.

a

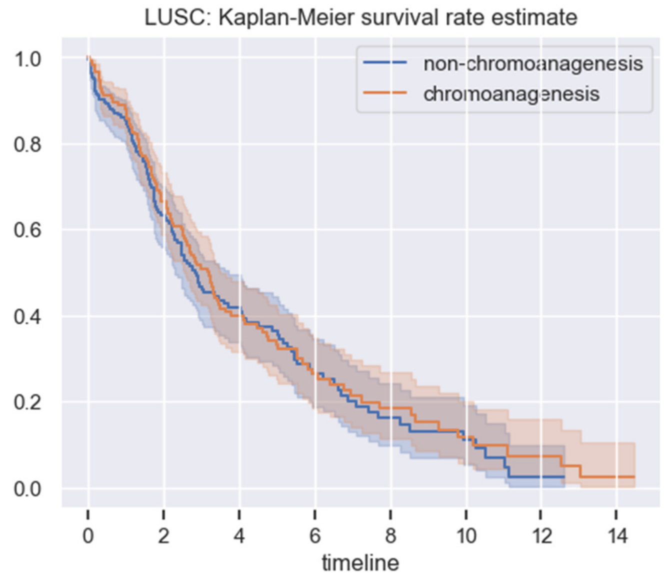

**chromoanagenesis**								
At risk	215	97	44	22	12	6	3	1
Censored	0	60	79	88	92	94	95	95
Events	0	58	92	105	111	115	117	119
**non-chromoanagenesis**								
At risk	218	96	48	23	12	6	1	0
Censored	0	55	73	82	85	88	89	90
Events	0	67	97	113	121	124	128	128

b

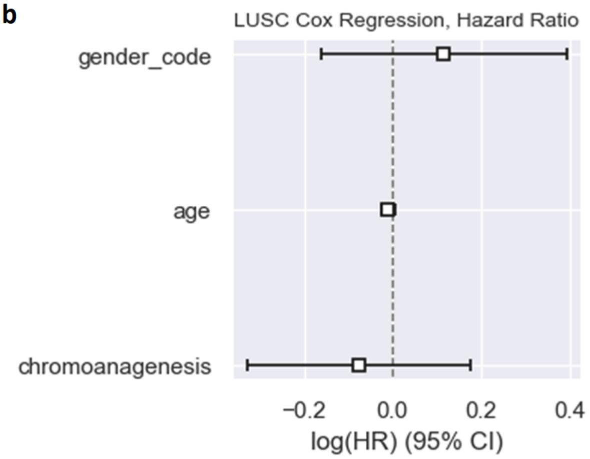

Figure S33. Kaplan-Meier Overall survival rate estimate for chromoanagenesis in OV.

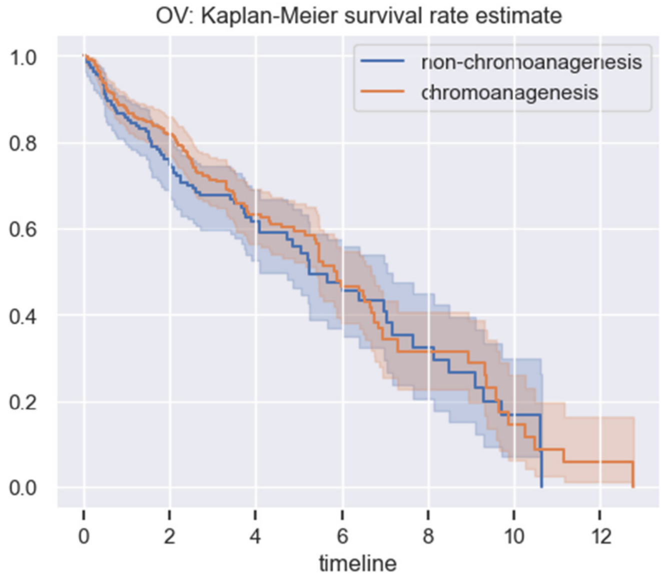

chromoanagenesis							
At risk	317	200	97	40	14	5	2
Censored	0	65	130	170	184	187	187
Events	0	52	90	107	119	125	128
non-chromoanagenesis							
At risk	188	109	51	24	11	4	0
Censored	0	36	78	96	103	105	107
Events	0	43	59	68	74	79	81

Figure S34. **(a)** Kaplan-Meier Overall survival rate estimate for chromoanagenesis in PAAD. **(b)** Cox regression hazard ratio, for chromoanagenesis, age and gender in PAAD patients.

a

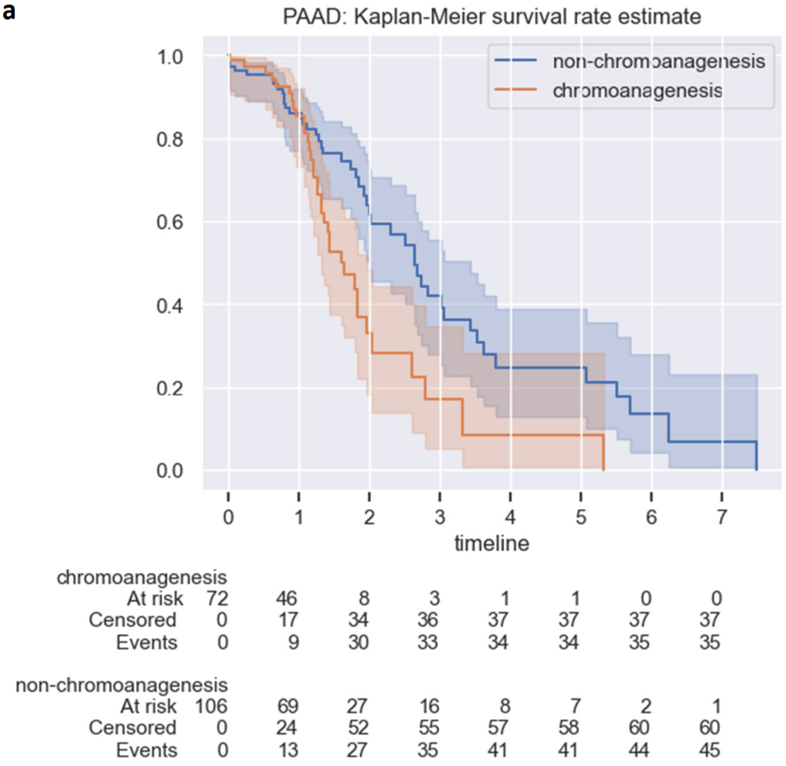

b

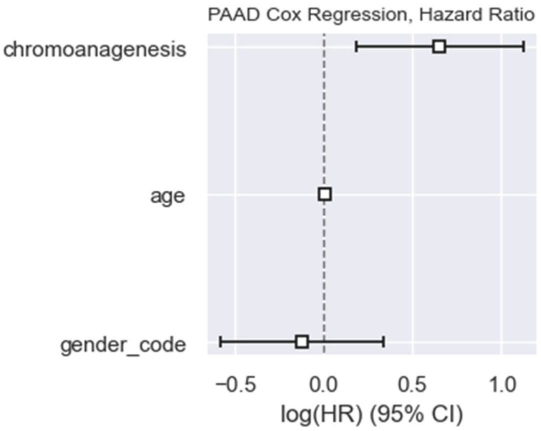

Figure S35. Kaplan-Meier Overall survival rate estimate for chromoanagenesis in PRAD.

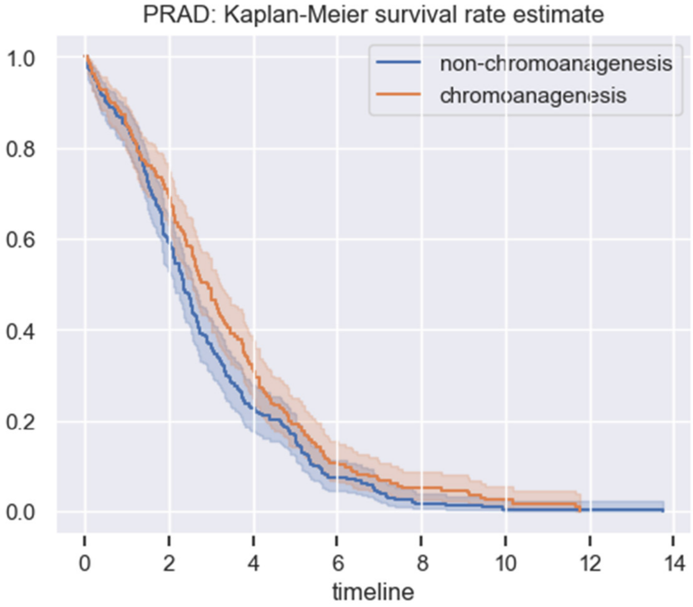

**chromoanagenesis**								
At risk	204	140	62	21	10	3	0	0
Censored	0	2	4	4	4	6	6	6
Events	0	62	138	179	190	195	198	198
**non-chromoanagenesis**								
At risk	254	148	56	18	4	1	1	0
Censored	0	3	4	5	5	5	5	5
Events	0	103	194	231	245	248	248	249

Figure S36. **(a)** Kaplan-Meier Overall survival rate estimate for chromoanagenesis in READ. **(b)** Cox regression hazard ratio, for chromoanagenesis, age and gender in READ patients.

a

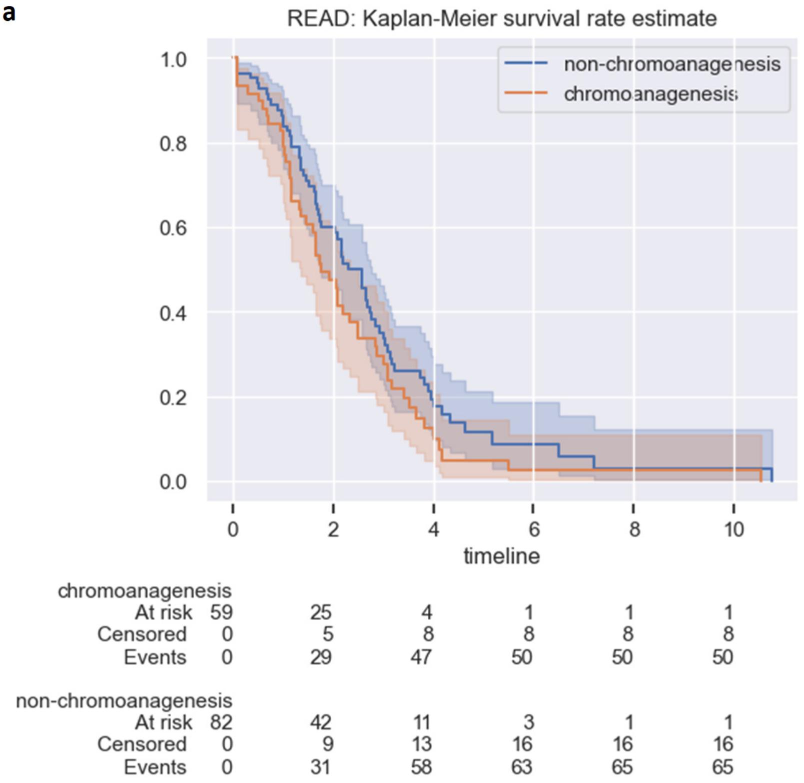

b

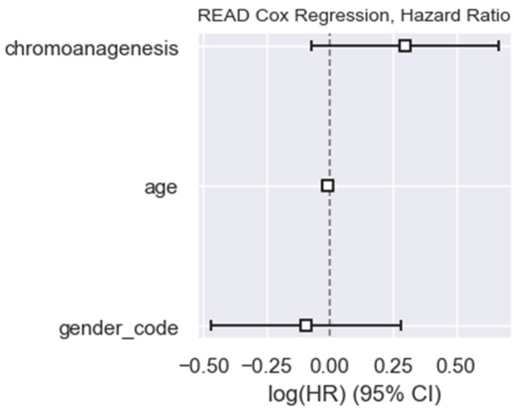

Figure S37. **(a)** Kaplan-Meier Overall survival rate estimate for chromoanagenesis in SARC. **(b)** Cox regression hazard ratio, for chromoanagenesis, age and gender in SARC patients.

a

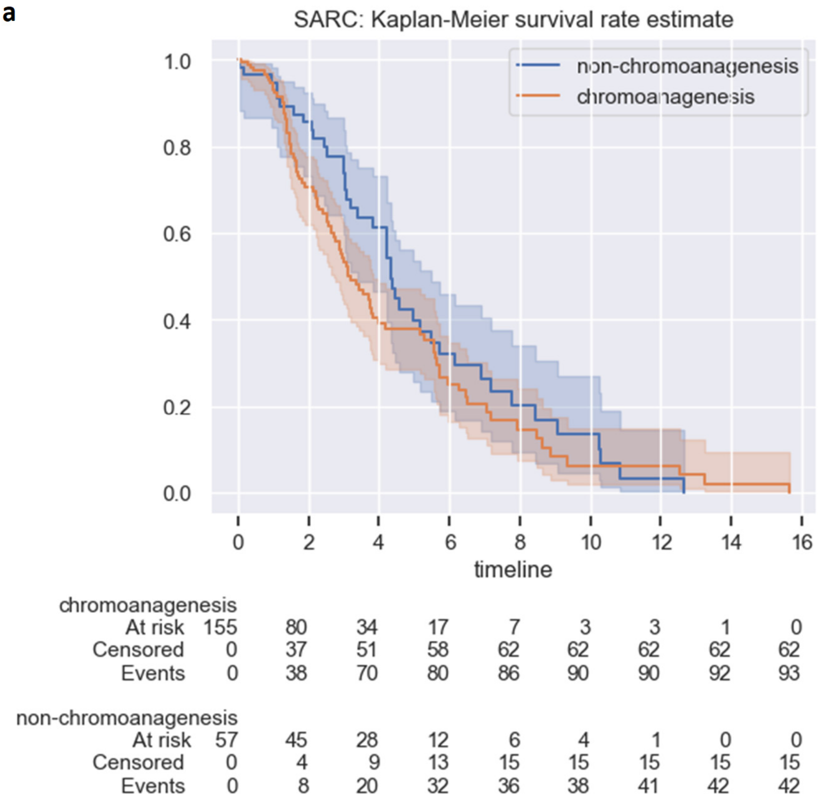

b

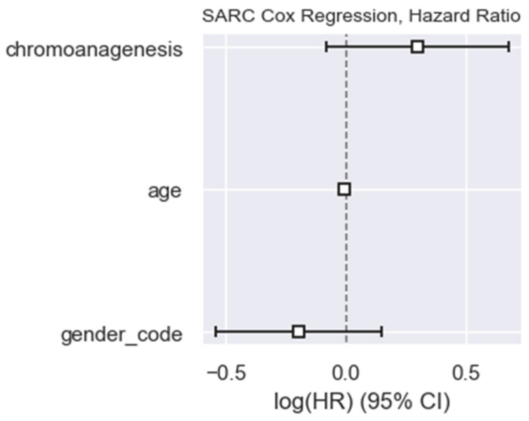

Figure S38. (a) Kaplan-Meier Overall survival rate estimate for chromoanagenesis in SKCM. **(b)** Cox regression hazard ratio, for chromoanagenesis, age and gender in SKCM patients.

a

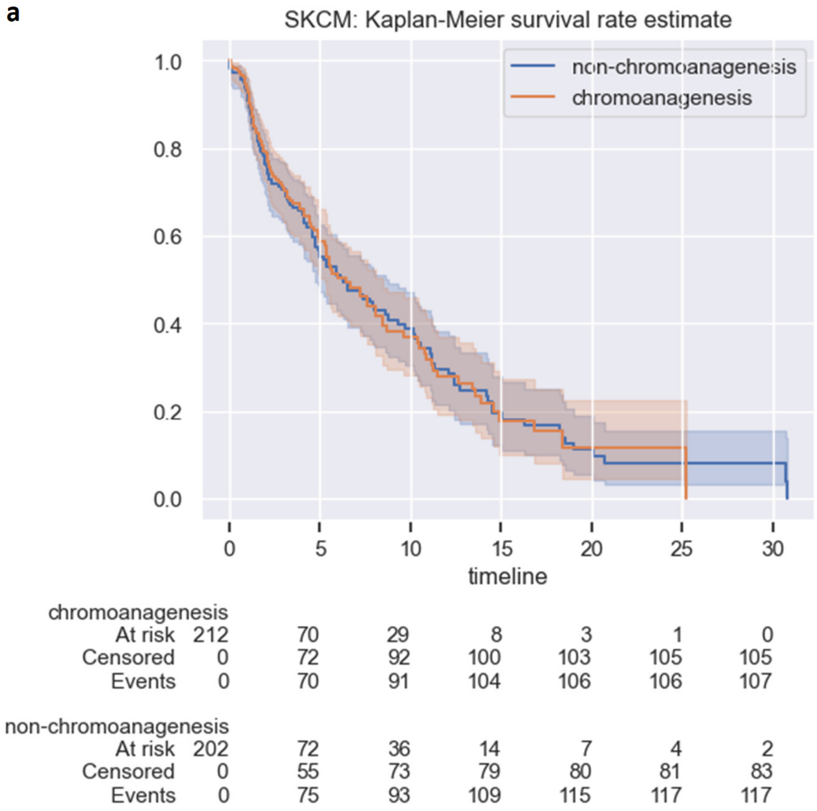

b

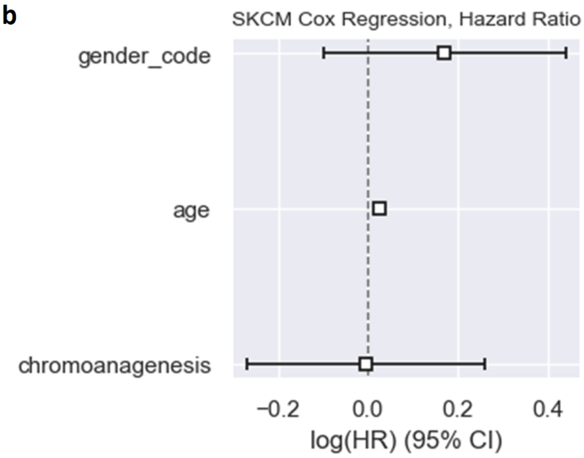

Figure S39. **(a)** Kaplan-Meier Overall survival rate estimate for chromoanagenesis in STAD. **(b)** Cox regression hazard ratio, for chromoanagenesis, age and gender in STAD patients.

a

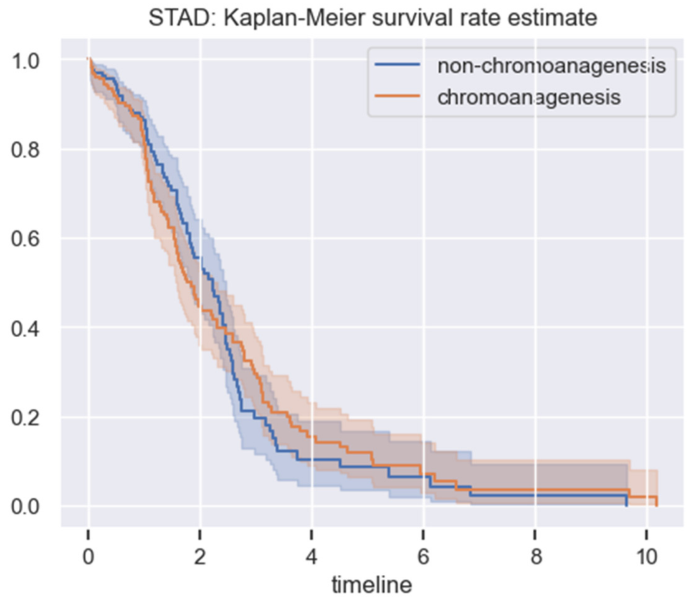

**chromoanagenesis**						
At risk	200	49	13	4	2	1
Censored	0	68	76	79	79	79
Events	0	83	111	117	119	120
**non-chromoanagenesis**						
At risk	166	48	6	3	1	0
Censored	0	67	76	77	77	77
Events	0	51	84	86	88	89

b

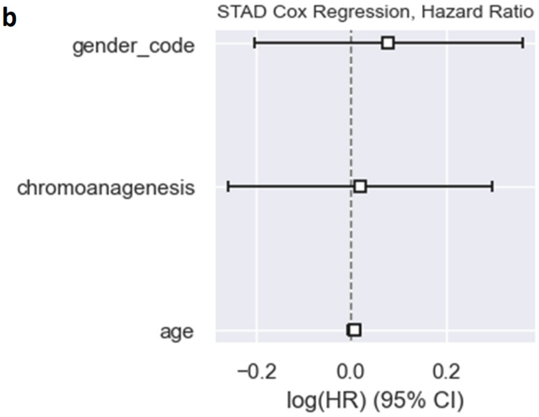

Figure S40. Kaplan-Meier Overall survival rate estimate for chromoanagenesis in UCEC.

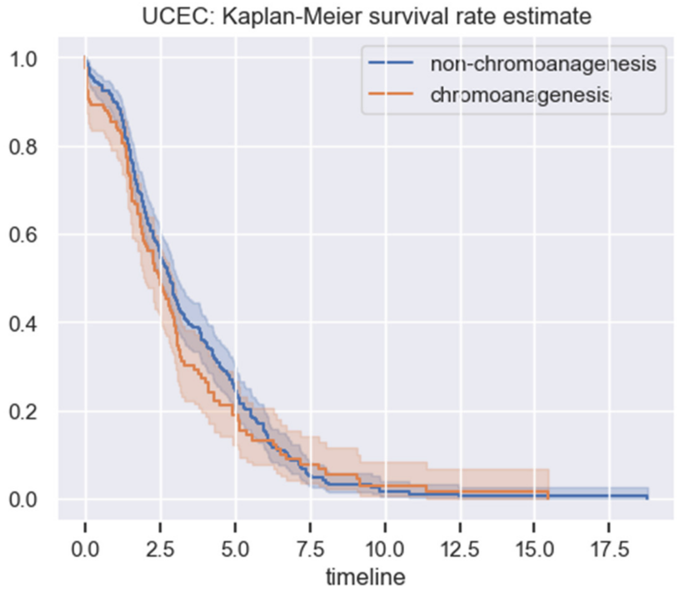

chromoanagenesis								
At risk	160	63	17	7	2	1	1	0
Censored	0	23	37	37	38	38	38	38
Events	0	74	106	116	120	121	121	122
non-chromoanagenesis								
At risk	324	163	69	13	3	1	1	1
Censored	0	25	32	35	37	37	37	37
Events	0	136	223	276	284	286	286	286
